# Supplementary material for: Cross species systems biology discovers glial DDR2, STOM, and KANK2 as therapeutic targets in progressive supranuclear palsy
Source: Nat Commun. 2023 Nov 2;14:6801. doi: 10.1038/s41467-023-42626-3 (PMC10622416; doi:10.1038/s41467-023-42626-3)
Supplement: Supplementary file 1 — Supplementary Information [file 41467_2023_42626_MOESM1_ESM.pdf]

Cross Species Systems Biology Discovers Glial *DDR2*, *STOM*, and *KANK2* as Therapeutic Targets in Progressive Supranuclear Palsy

Supplementary Figures and Tables

Yuhao Min[1,2,3], Xue Wang[4], Özkan İş[1], Tulsi A. Patel[1], Junli Gao[1], Joseph S. Reddy[4], Zachary S. Quicksall[4], Thuy Nguyen[1], Shu Lin[1], Frederick Q. Tutor-New[1], Jessica L. Chalk[1], Adriana O. Mitchell[1], Julia E. Crook[2], Peter T. Nelson[5,6], Linda J. Van Eldik[5,7], Todd E. Golde[8], Minerva M. Carrasquillo[1], Dennis W. Dickson[1], Ke Zhang[1], Mariet Allen[1], Nilüfer Ertekin-Taner[1,9#]

Author Affiliations:

1. Department of Neuroscience, Mayo Clinic, Jacksonville, Florida, USA
2. Center for Clinical and Translational Science, Mayo Clinic, Rochester, Minnesota, USA
3. Mayo Clinic Graduate School of Biomedical Sciences, Mayo Clinic, Jacksonville, Florida, USA
4. Department of Quantitative Health Sciences, Mayo Clinic, Jacksonville, Florida, USA
5. Sanders-Brown Center on Aging, University of Kentucky, Lexington, Kentucky, USA
6. Department of Pathology & Laboratory Medicine, University of Kentucky, Lexington, Kentucky, USA
7. Department of Neuroscience, University of Kentucky, Lexington, Kentucky, USA
8. Department of Pharmacology and Chemical Biology, Department of Neurology, Emory Center for Neurodegenerative Disease, Emory University, Atlanta, Georgia, USA
9. Department of Neurology, Mayo Clinic, Jacksonville, Florida, USA

# Corresponding author: taner.nilufer@mayo.edu

Figure S1 .... **4**
Figure S2 .... **5**
Figure S3 .... **6**
Figure S4 .... **7**
Figure S5 .... **8**
Figure S6 .... **9**
Figure S7 .... **10**
Figure S8 .... **11**
Figure S9 .... **13**
Figure S10 .... **14**
Figure S11 .... **15**
Figure S12 .... **16**
Figure S13 .... **17**
Figure S14 .... **18**
Figure S15 .... **19**

Figure S16 .... *20*
Figure S17 .... *23*
Figure S18 .... *25*
Figure S19 .... *26*
Figure S20 .... *27*
Figure S21 .... *28*
Table S1 .... *29*
Table S2 .... *30*
Table S3 .... *31*
Table S4 .... *32*
Table S5 .... *33*
Table S6 .... *34*
Table S7 .... *35*
Table S8 .... *36*
Table S9 .... *37*
Table S10 .... *38*

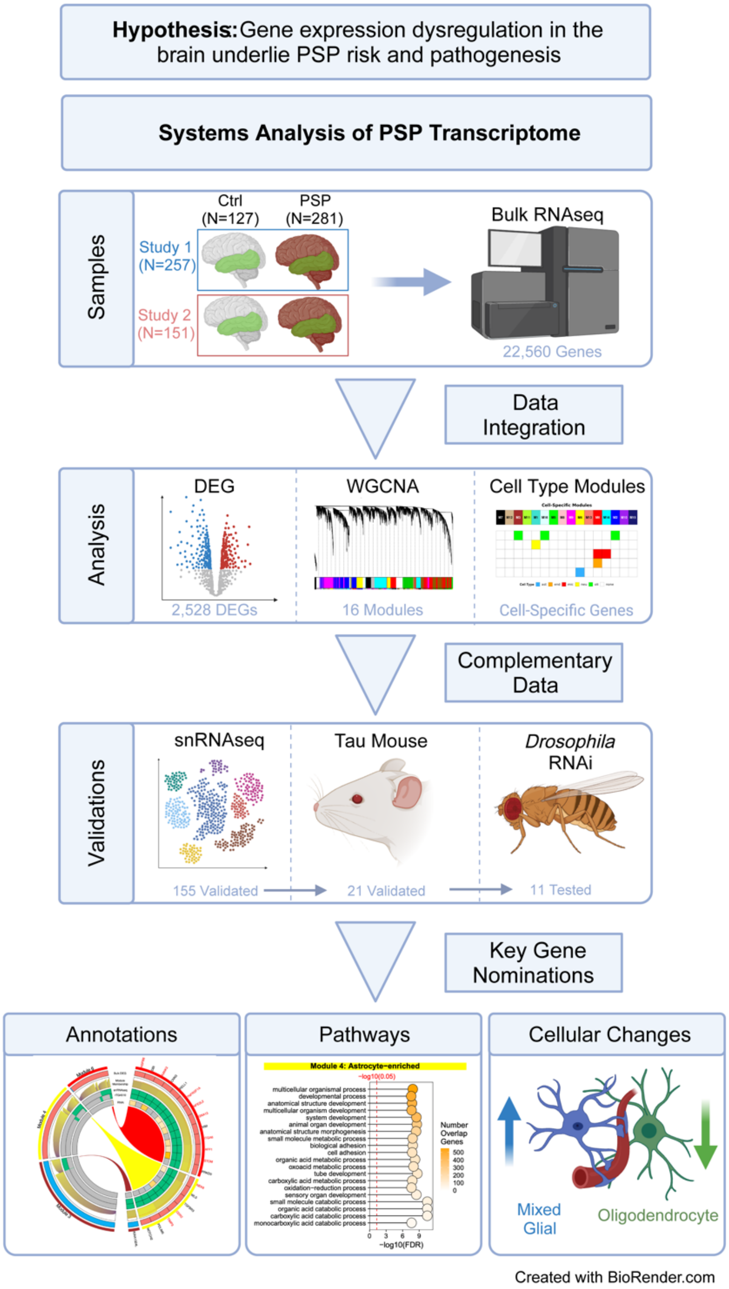

Figure S-i. Graphic abstract depicting the key steps and the overall workflow of this manuscript.

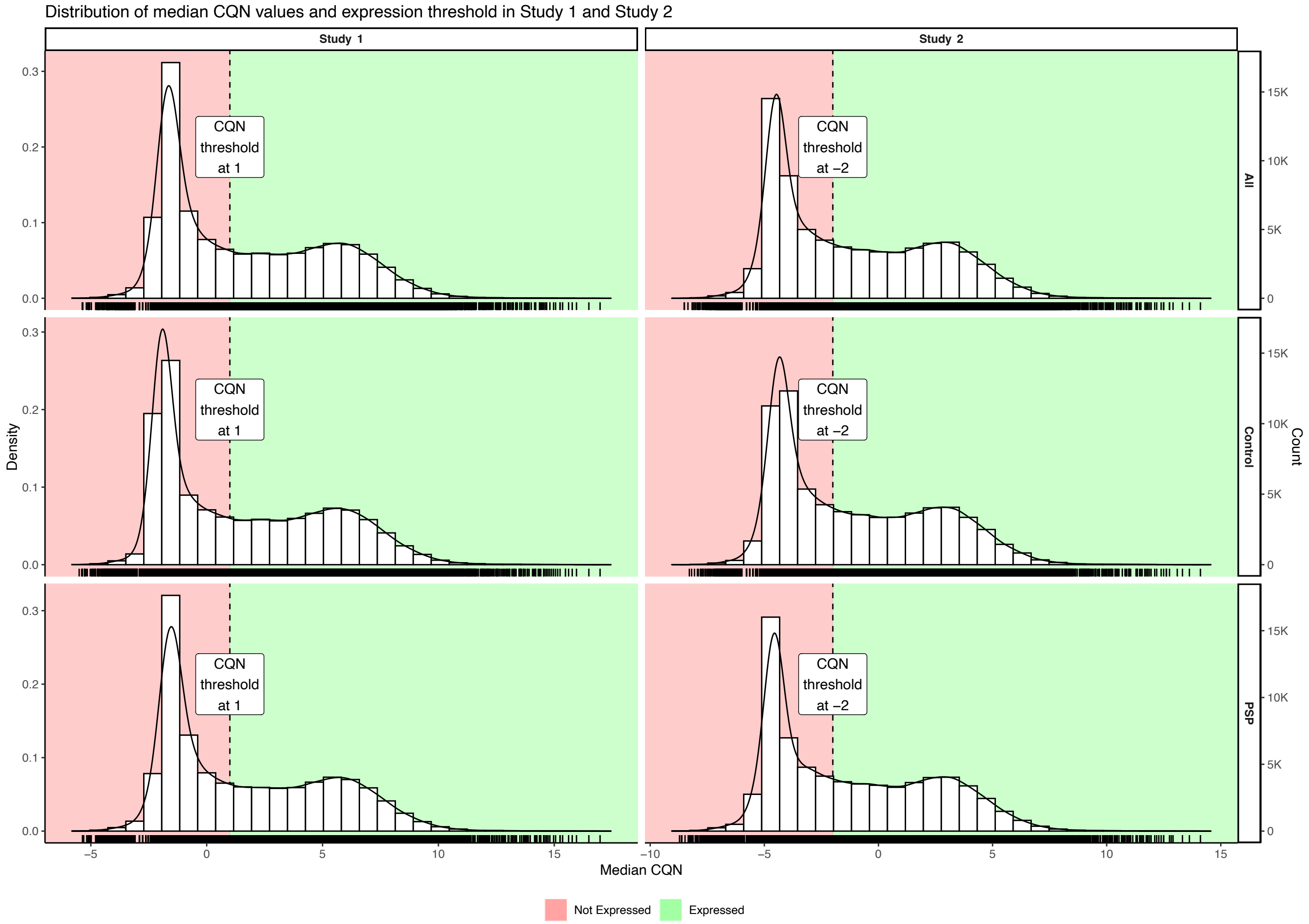

Figure S1. Distribution of the median conditional quantile normalization (CQN) value for each gene (Total genes = 54,991 before filtering) in the two bulk RNAseq cohorts. Based on the bimodal distribution, expression thresholds were chosen at 1 and -2 (dotted lines), respectively. Source data are provided as a Source Data file.

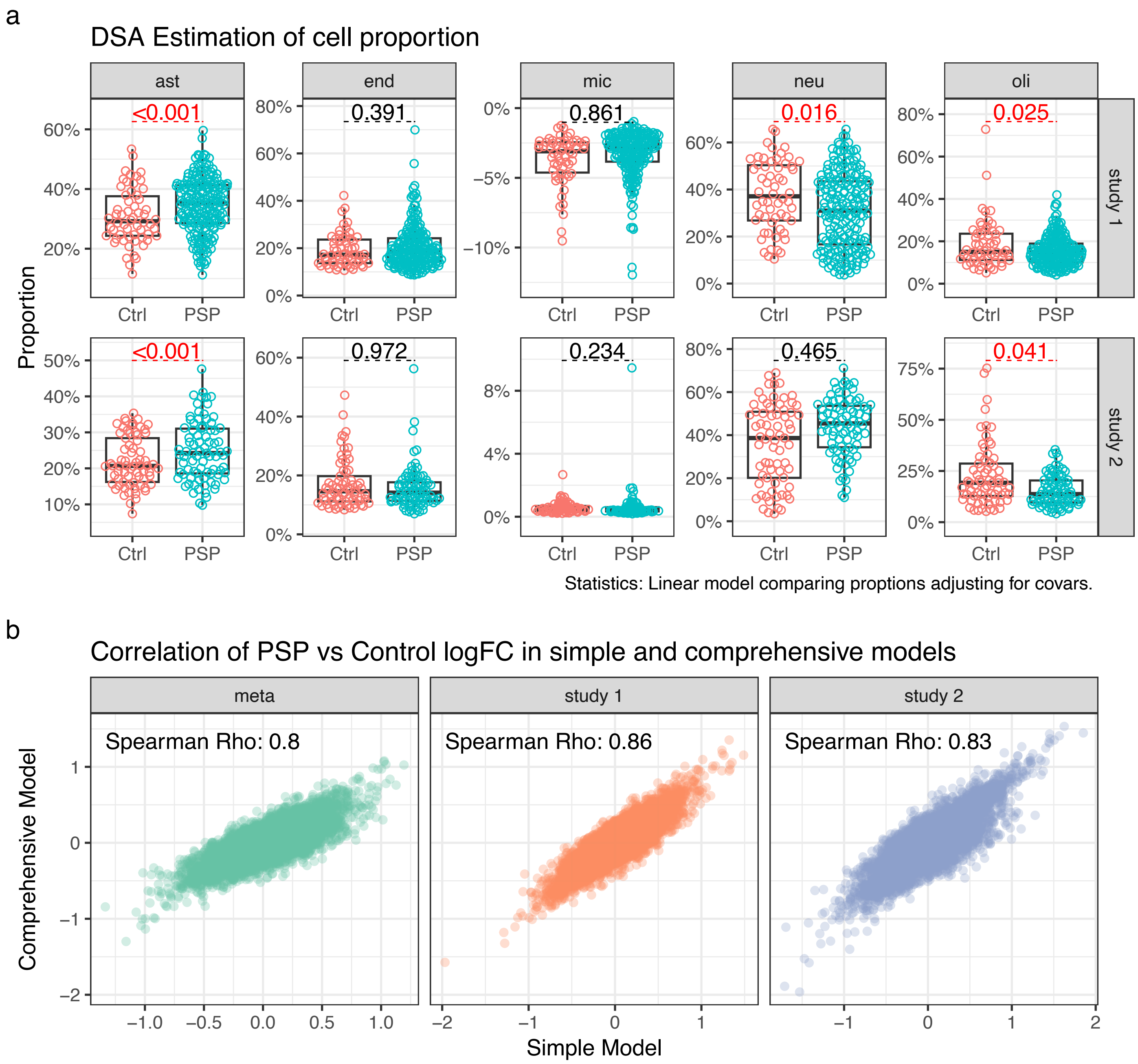

Figure S2. Secondary analysis of the bulk RNAseq data to assess cell proportion changes in PSP. **a**: Estimated cell proportions in PSP and control participants using the Digital Sorting Algorithm. Study 1: N = 257 PSP and Control individuals; Study 2: N = 151 PSP and Control individuals. **b**: Correlation between the PSP vs Control logFC derived from the Simple model (not adjusting for cell proportion) and the Comprehensive model (adjusting for cell proportion). N = 22,560 genes. Source data are provided as a Source Data file.

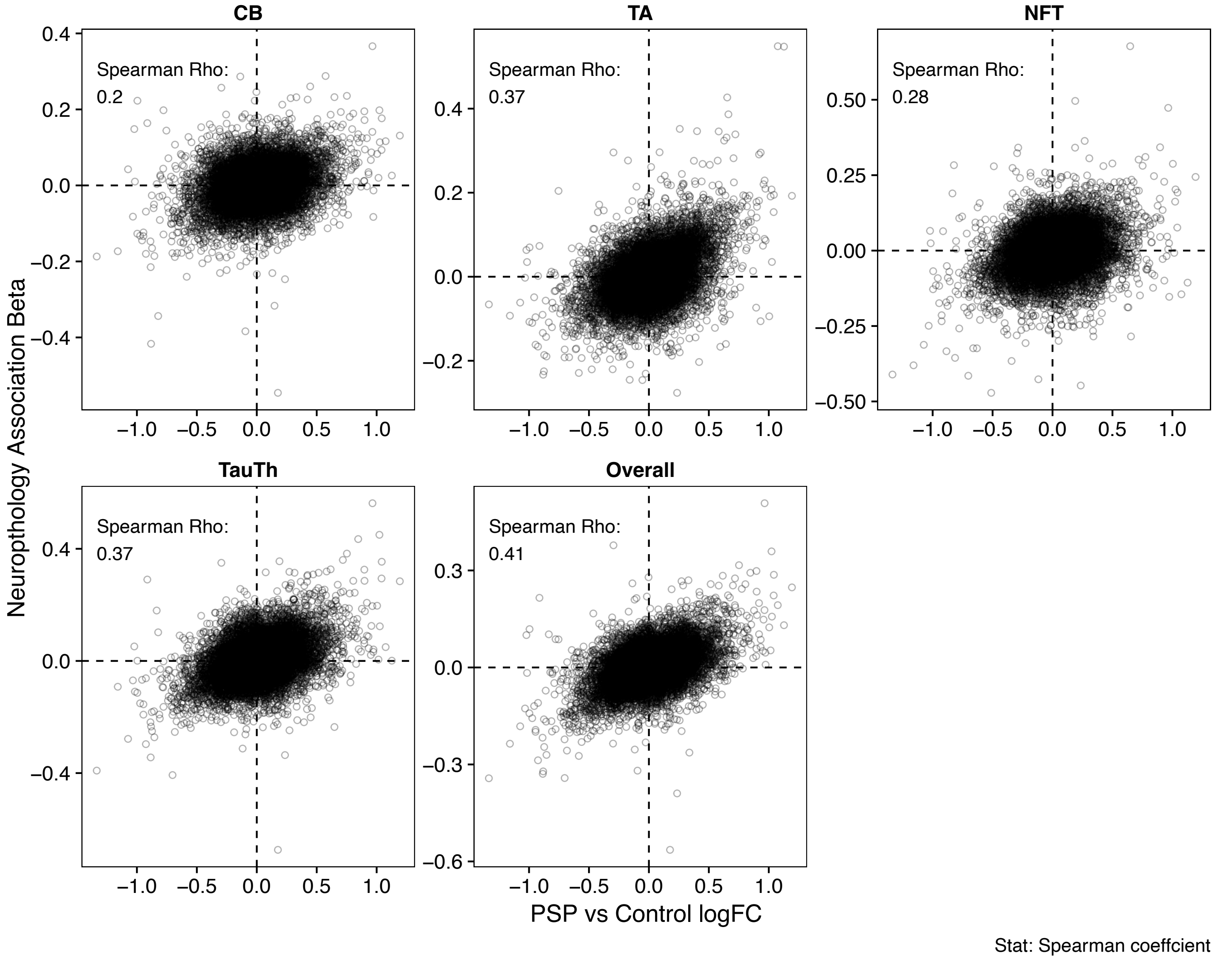

Figure S3. Correlation between beta coefficients from PSP vs Control DEG and those from gene expression associations with quantitative tau neuropathology in PSP. Statistics: Spearman correlation. N = 22,560 genes. Source data are provided as a Source Data file.

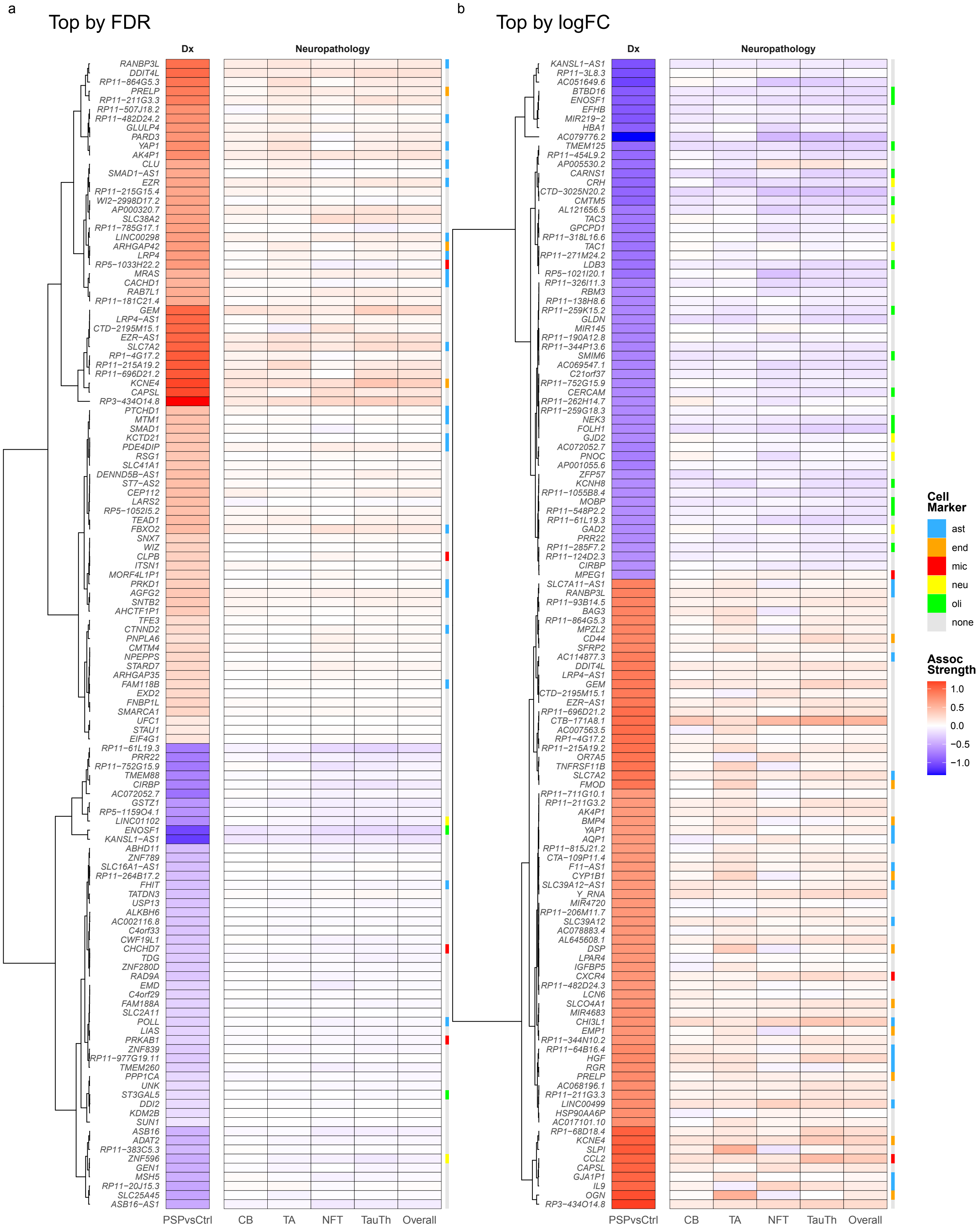

Figure S4. Heatmaps of bulk DEGs between PSP and control brains that represent **a** Lowest 5% FDR (most statistically significant) or **b** highest |logFC| (largest expression difference). Source data are provided as a Source Data file.

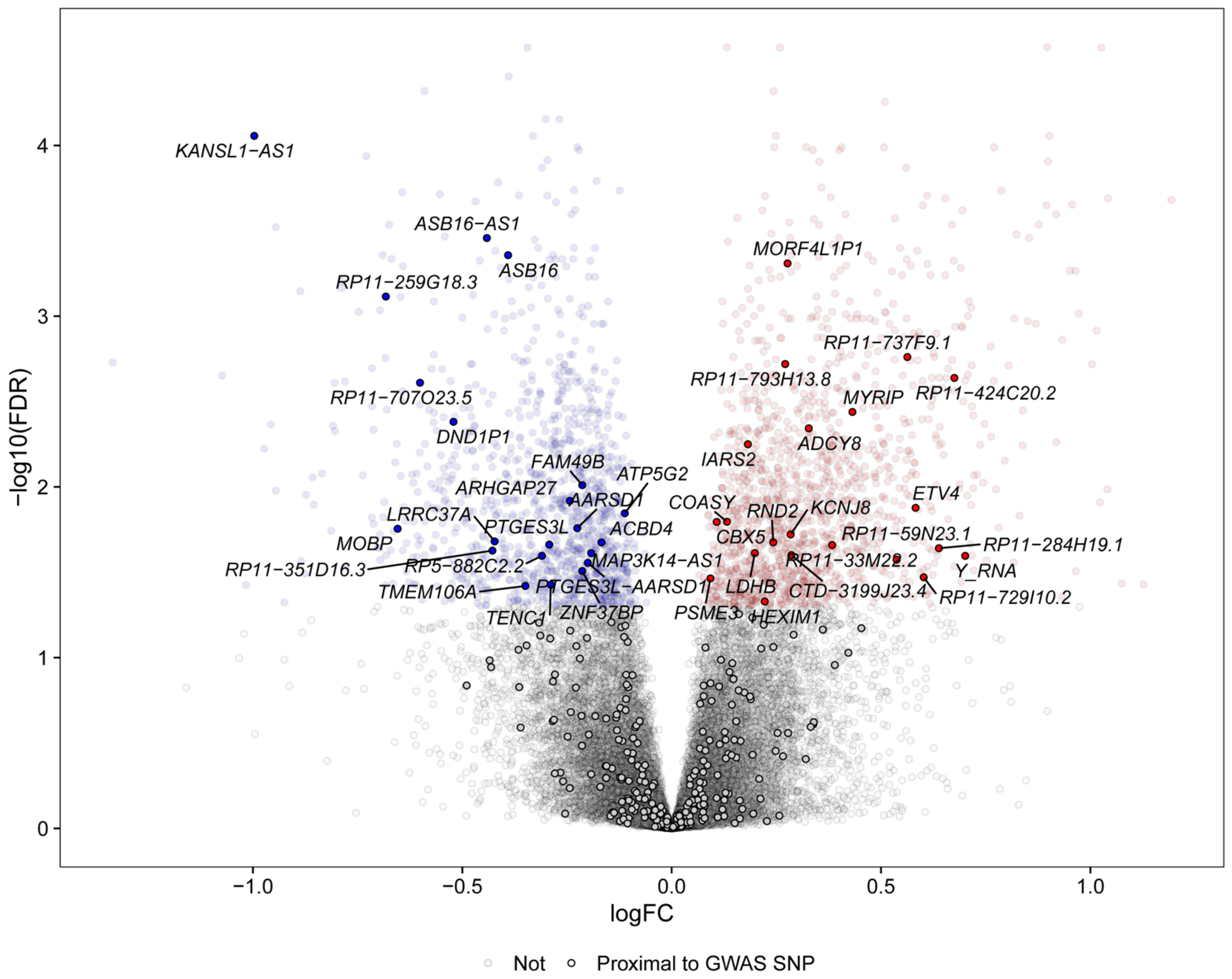

Figure S5. Volcano plot highlighting DEGs between PSP and control that are within 1 Mbp of PSP genetic risk loci from previous GWAS studies. Red dots: logFC > 0 and FDR <0.05, blue dots: logFC < 0 and FDR <0.05, gray: FDR >0.05. Source data are provided as a Source Data file.

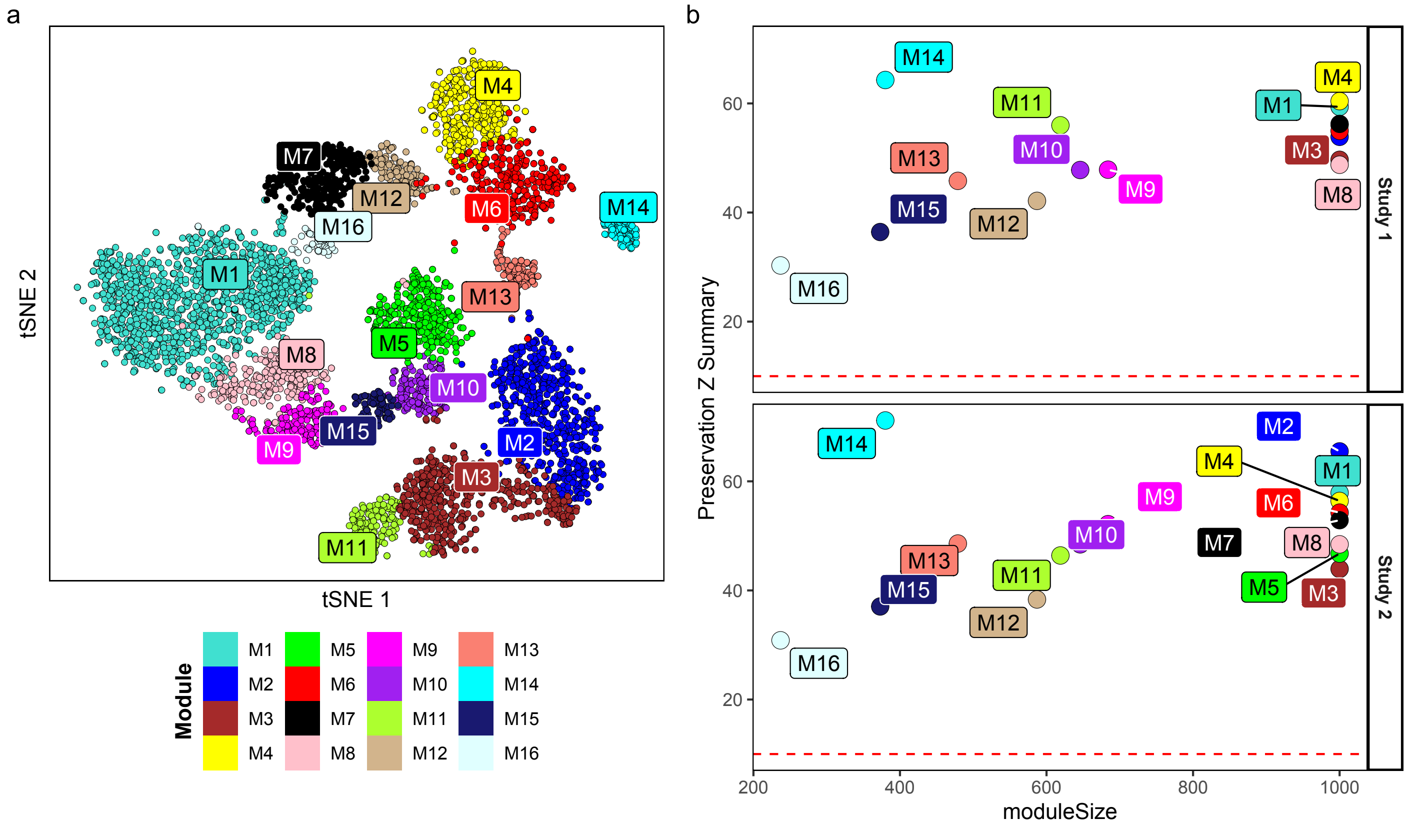

Figure S6. The consensus WGCNA network definition is robust. **a**: Independent embedding of the expression data in tSNE space showed high agreement between the gene cluster and module assignment. **b**: Module preservation analysis indicating all modules are well-preserved with a summary score above the threshold (10, red dotted line). Source data are provided as a Source Data file.

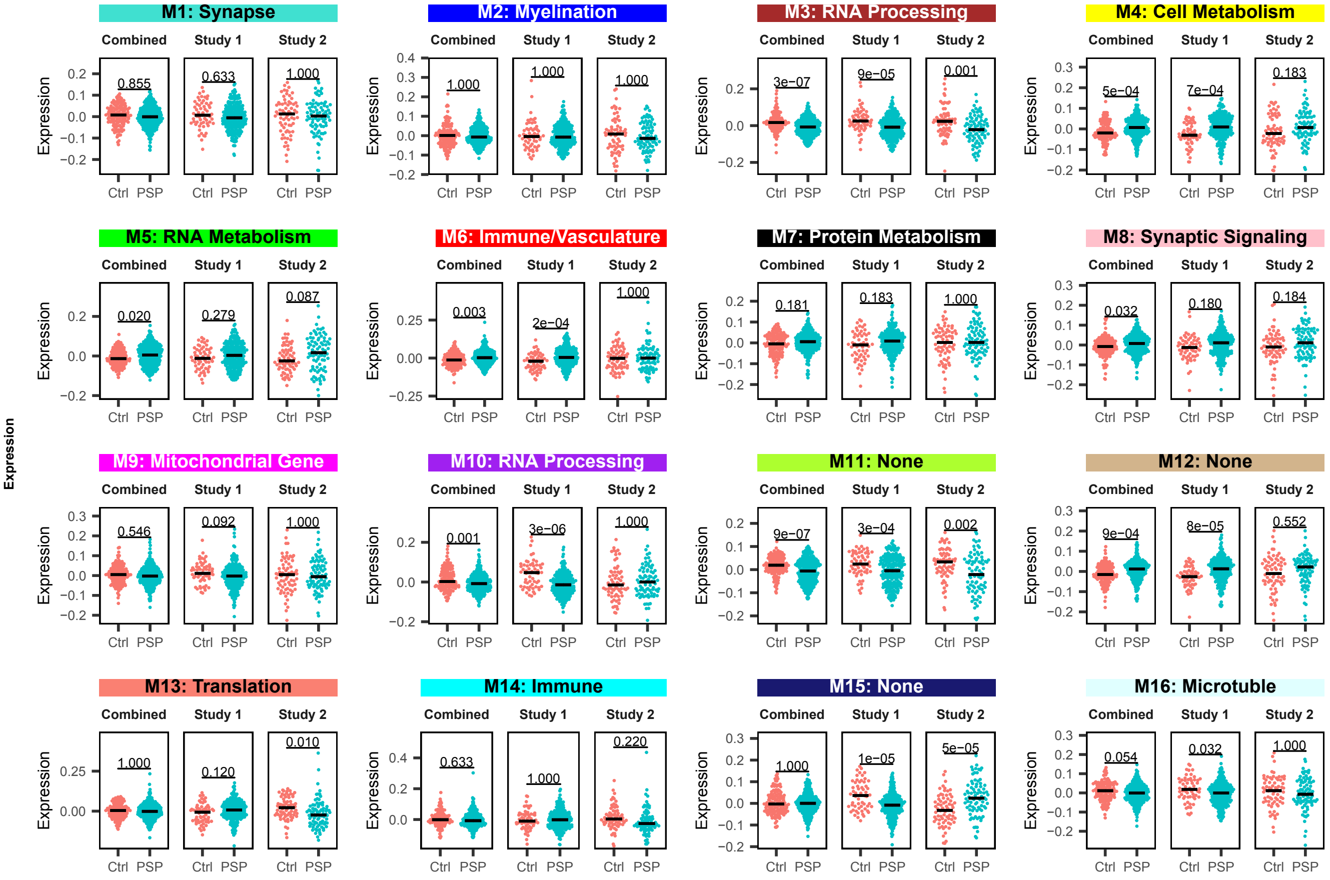

*bonferroni adjusted p value for t−test

Figure S7. Module eigengene distribution between PSP and Control samples in study 1, study 2, and the consensus network for all 16 modules. Statistics: two-sided t-test comparing PSP and control module eigengenes. Bonferroni- adjusted p values are presented. Study 1: N = 257 PSP and Control individuals; Study 2: N = 151 PSP and Control individuals; Combined: N= 408 PSP and Control individuals.

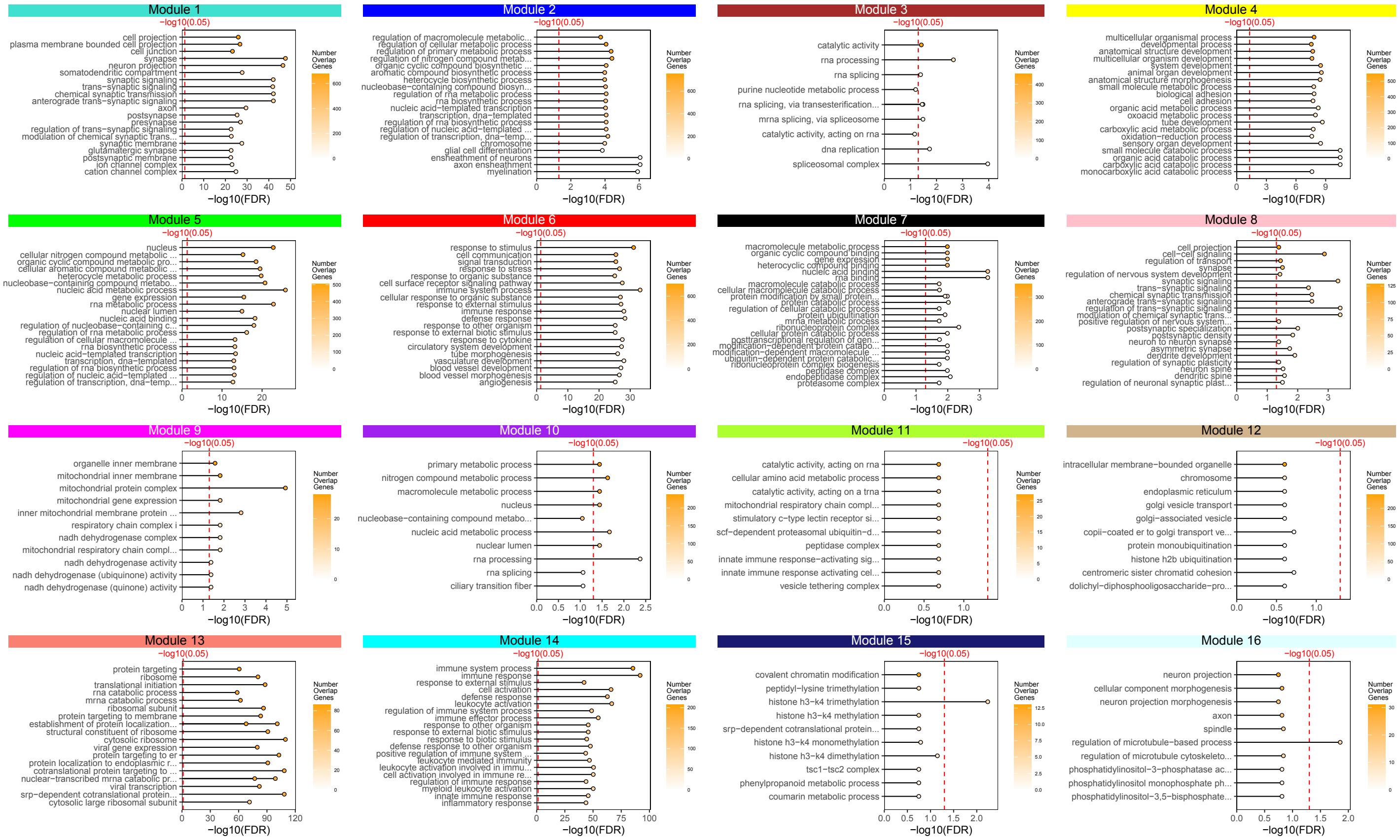

Figure S8. Top enriched Gene Ontology Biological Process terms in each of the WGCNA modules. Source data are provided as a Source Data file.

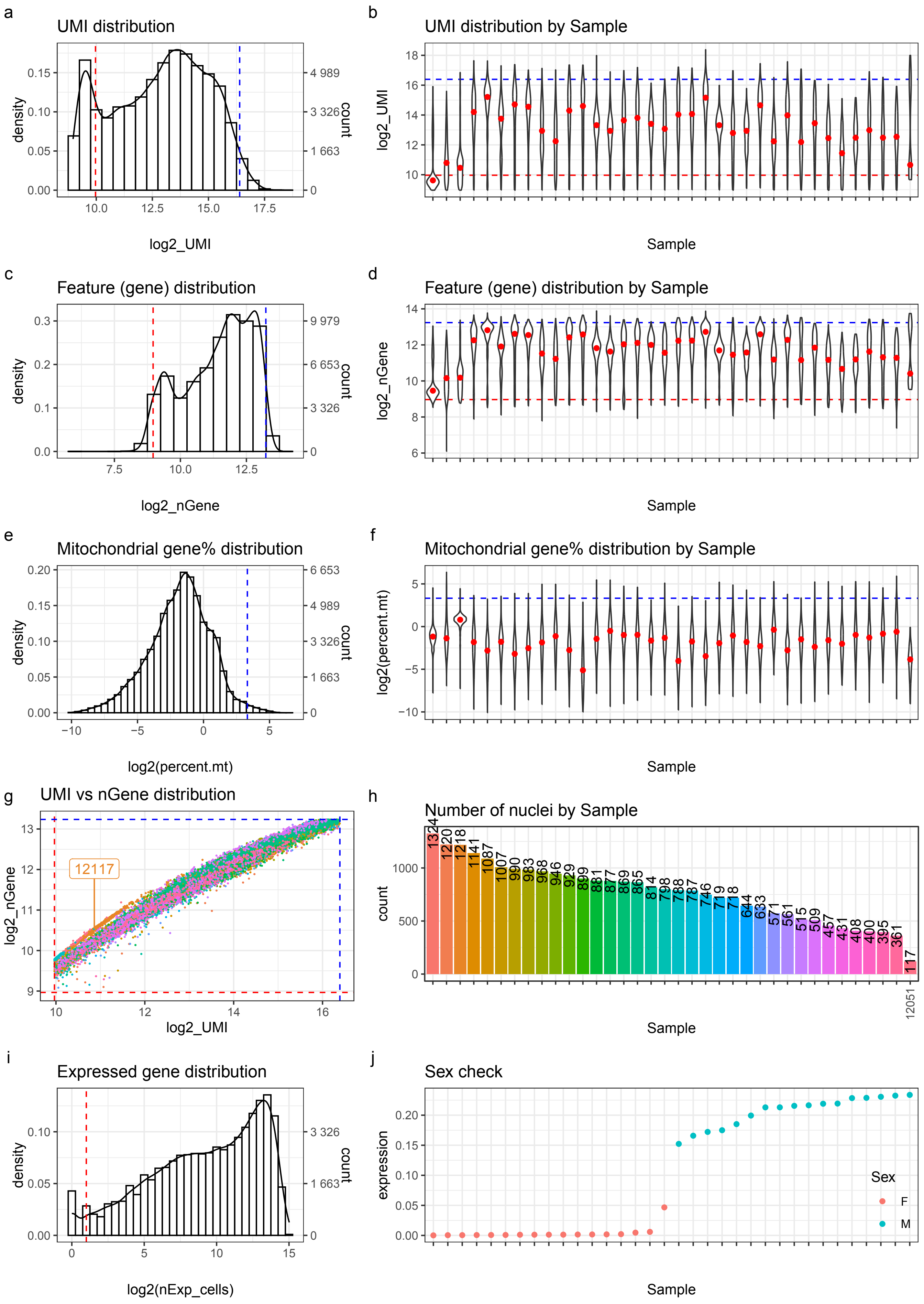
a
UMI distribution
density
count
log2_UMI
b
UMI distribution by Sample
log2_UMI
Sample
c
Feature (gene) distribution
density
count
log2_nGene
d
Feature (gene) distribution by Sample
log2_nGene
Sample
e
Mitochondrial gene% distribution
density
count
log2(percent.mt)
f
Mitochondrial gene% distribution by Sample
log2(percent.mt)
Sample
g
UMI vs nGene distribution
12117
log2_nGene
log2_UMI
h
Number of nuclei by Sample
1324 1220 1218 1141 1087 1007 990 983 968 946 929 899 881 877 869 865 814 798 788 787 746 719 718 644 633 571 561 515 509 457 431 408 400 395 361 117
count
12051
Sample
i
Expressed gene distribution
density
count
log2(nExp_cells)
j
Sex check
expression
Sex
F
M
Sample

Figure S9. Quality-Control metrics for the snRNAseq analysis. **a-b**: Distribution of the number of mapped UMIs from all nuclei and in each sample. **c-d**: Distribution of the number of detected genes from all nuclei and in each sample. **e-f**: Distribution of the percent of mitochondrial genes from all nuclei and in each sample. **g**: Distribution of the number of mapped UMI vs the number of detected genes in each sample. **h**: Number of nuclei in each sample. **i**: Distribution of the number of nuclei that are expressing each gene. **j**: Expression of chromosome Y genes *RPS4Y1*, *EIF1AY*, *DDX3Y*, and *KDM5D*. Source data are provided as a Source Data file.

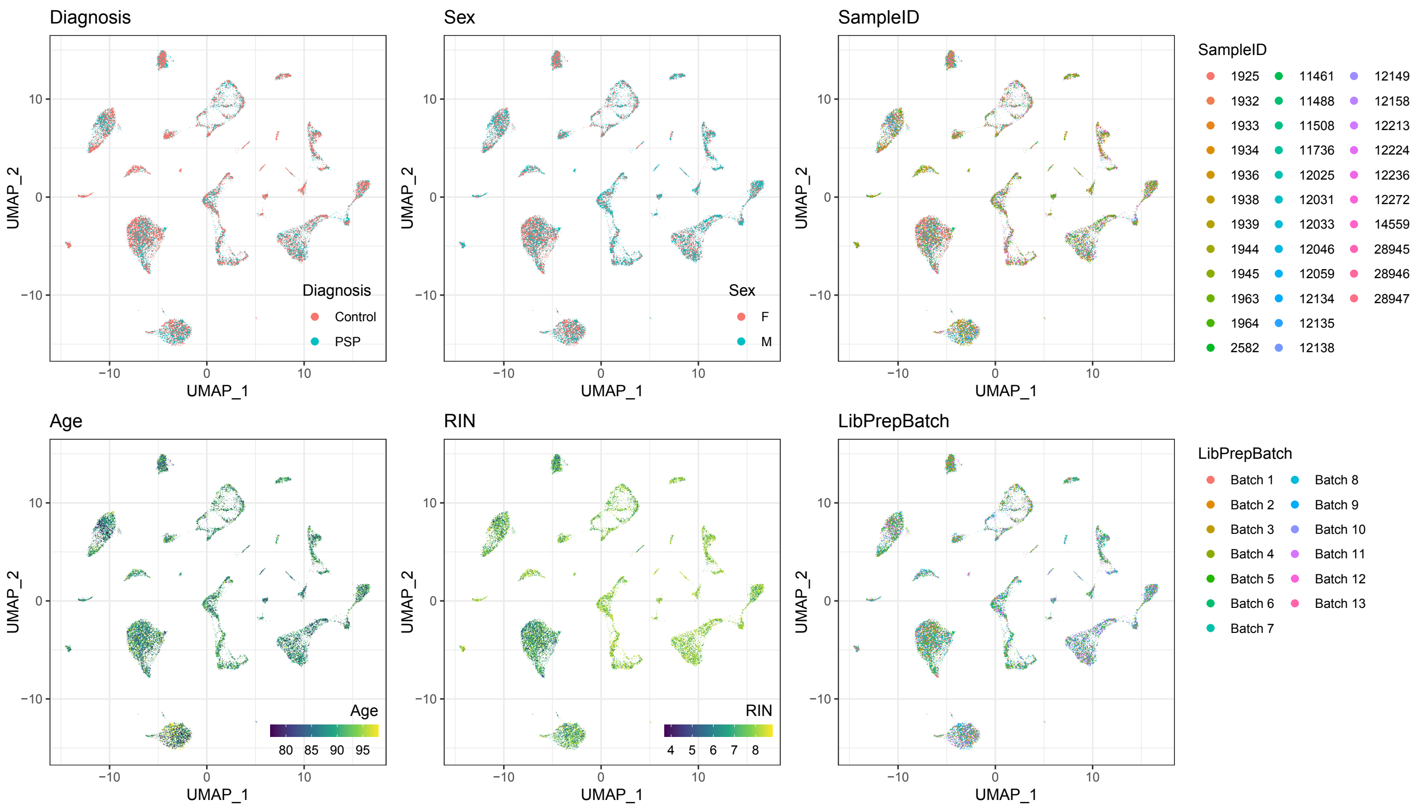

Figure S10. Nuclei in UMAP space colored by different variables.

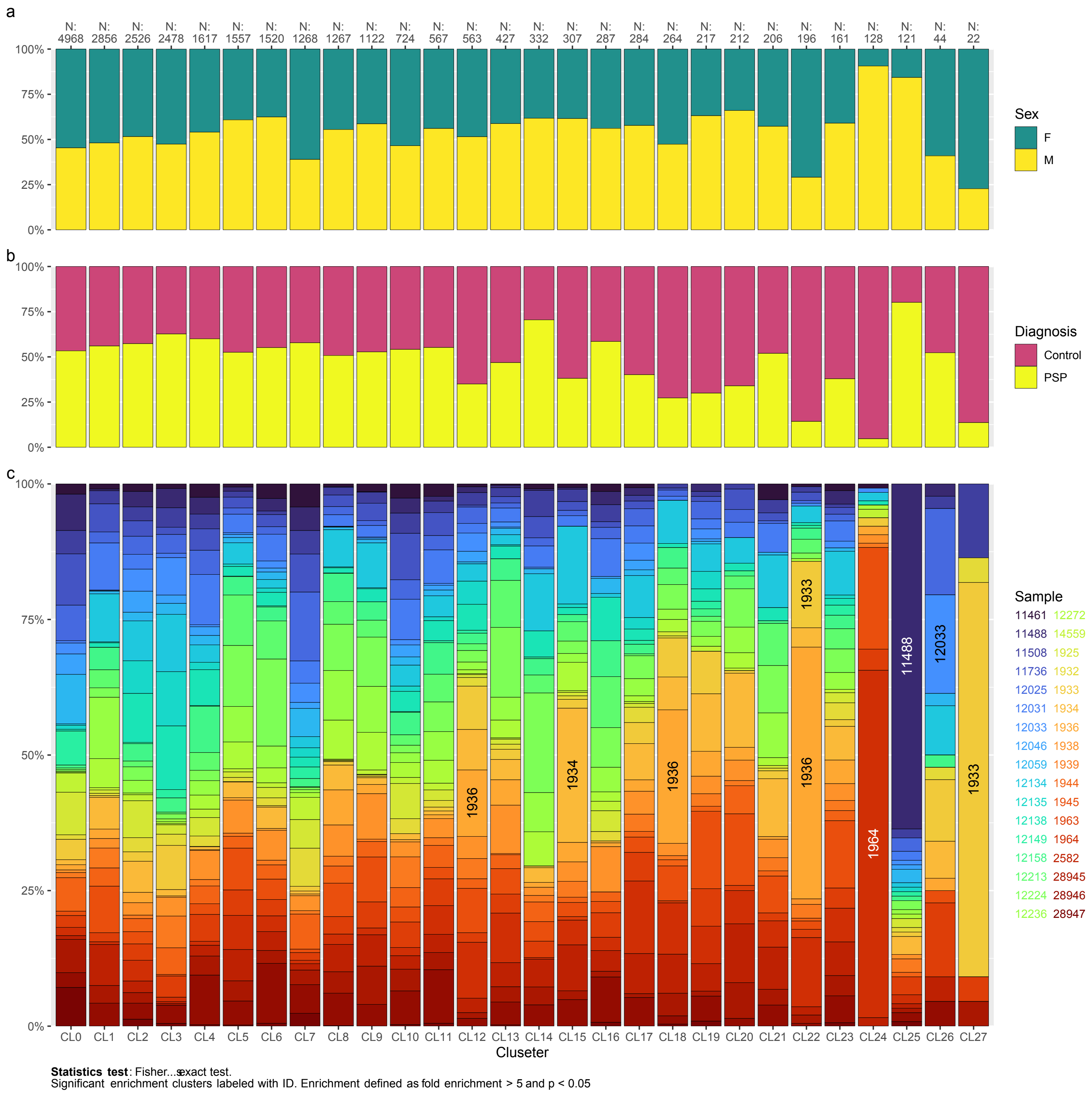

Figure S11. Stacked bar plots show the proportion of nuclei from different **a**: sex, **b**: diagnosis, and **c**: sample in each snRNAseq cluster. There was no statistically significant enrichment of nuclei from either sex or diagnosis in any of the clusters. Eight clusters had statistically significant enrichment of nuclei from a small number of samples, depicted with sample numbers on the clusters. All but one of these 8 clusters (CL12) constitute <1% of total nuclei. Statistics: one-sided Fisher Exact test. Source data are provided as a Source Data file.

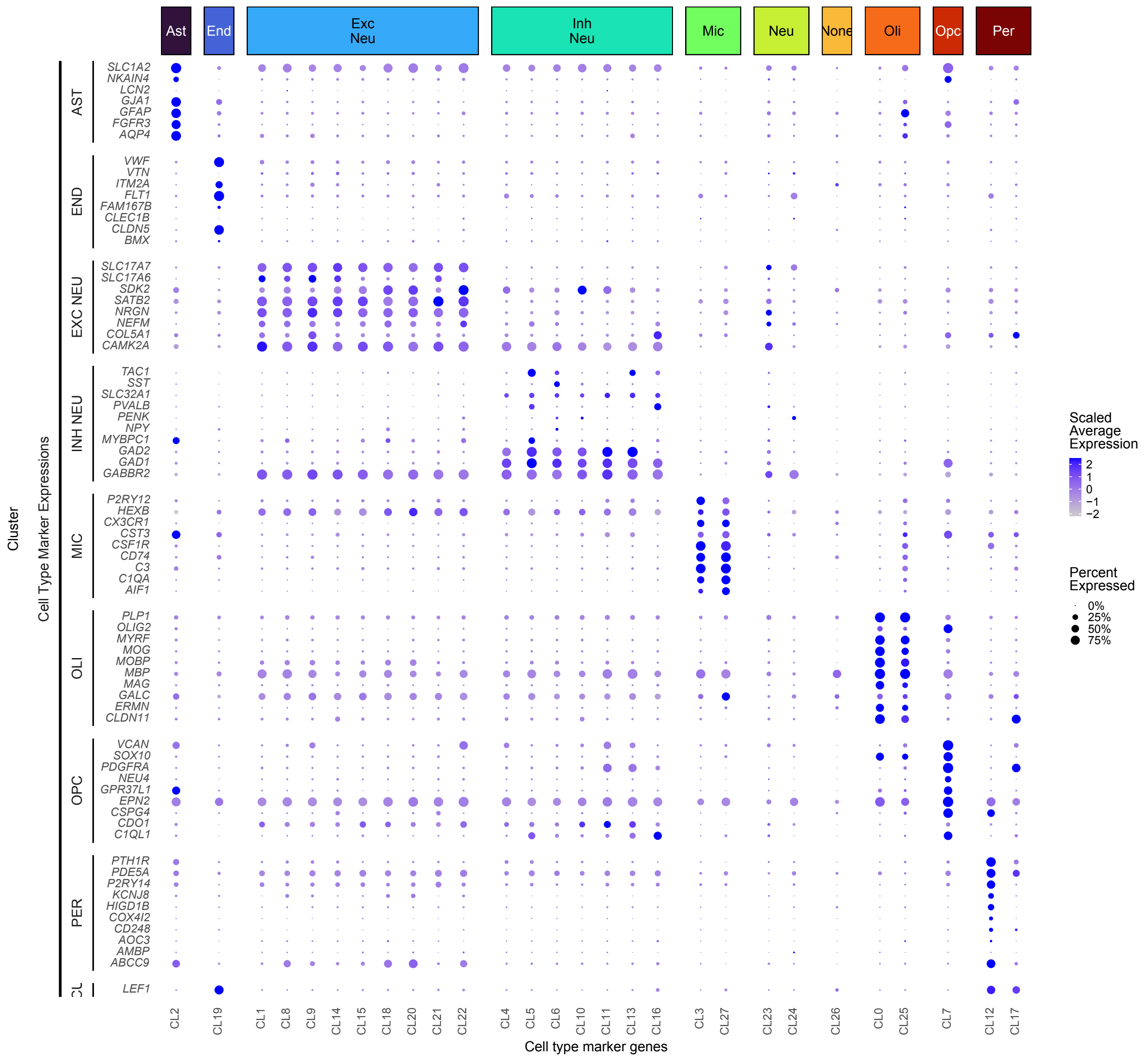

Figure S12. Expression of an extended external list[1] of cell-type marker genes in each snRNAseq cluster confirmed our cluster identities. Source data are provided as a Source Data file.

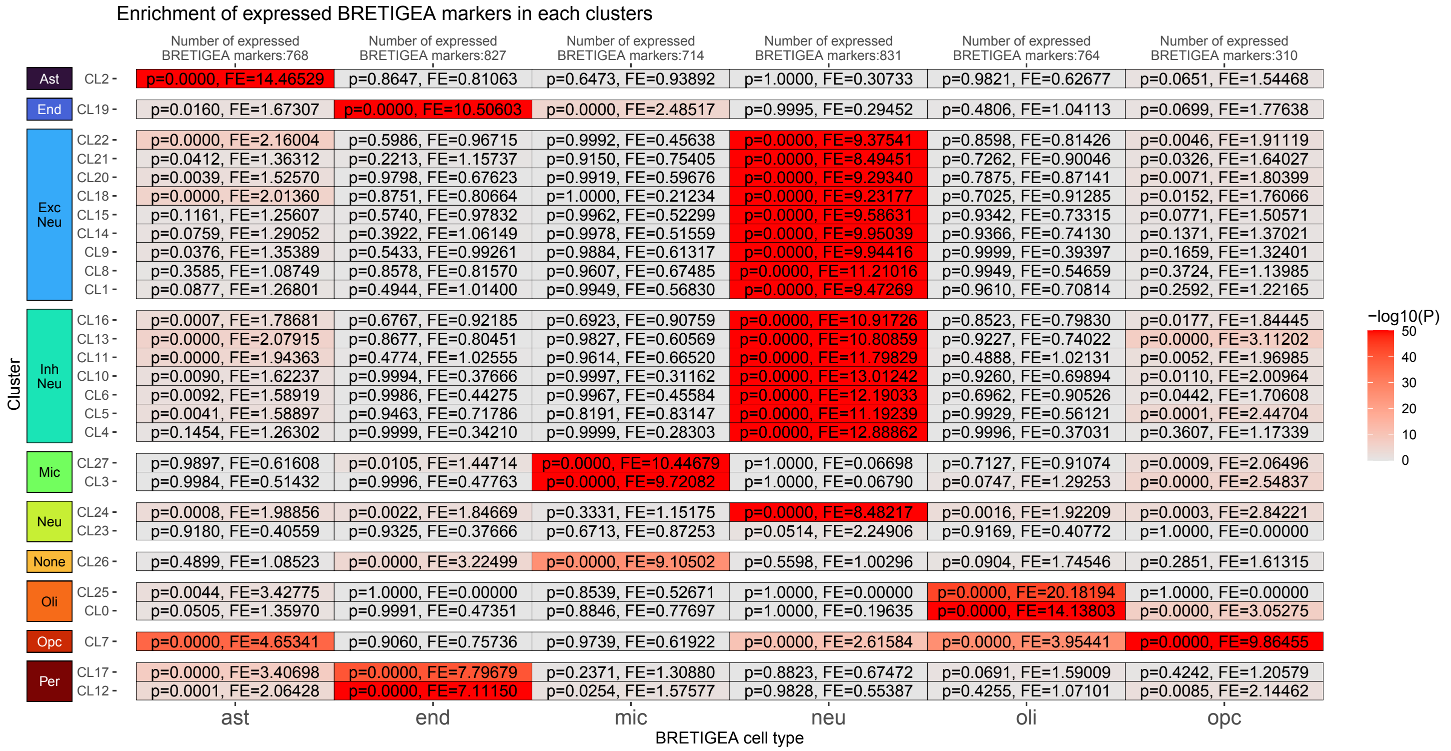

Figure S13. Enrichment of BRETIGEA[2] cell-type marker genes in each cluster corresponds to the cluster type. BRETIGEA cell-types for each column are shown at the bottom and number of BRETIGEA genes for these cell-types expressed in our snRNAseq data is shown at the top. P value and fold enrichment of these BRETIGEA cell-type marker genes for each cluster are shown in the data cells. Statistics: one-sided Fisher Exact test. Source data are provided as a Source Data file.

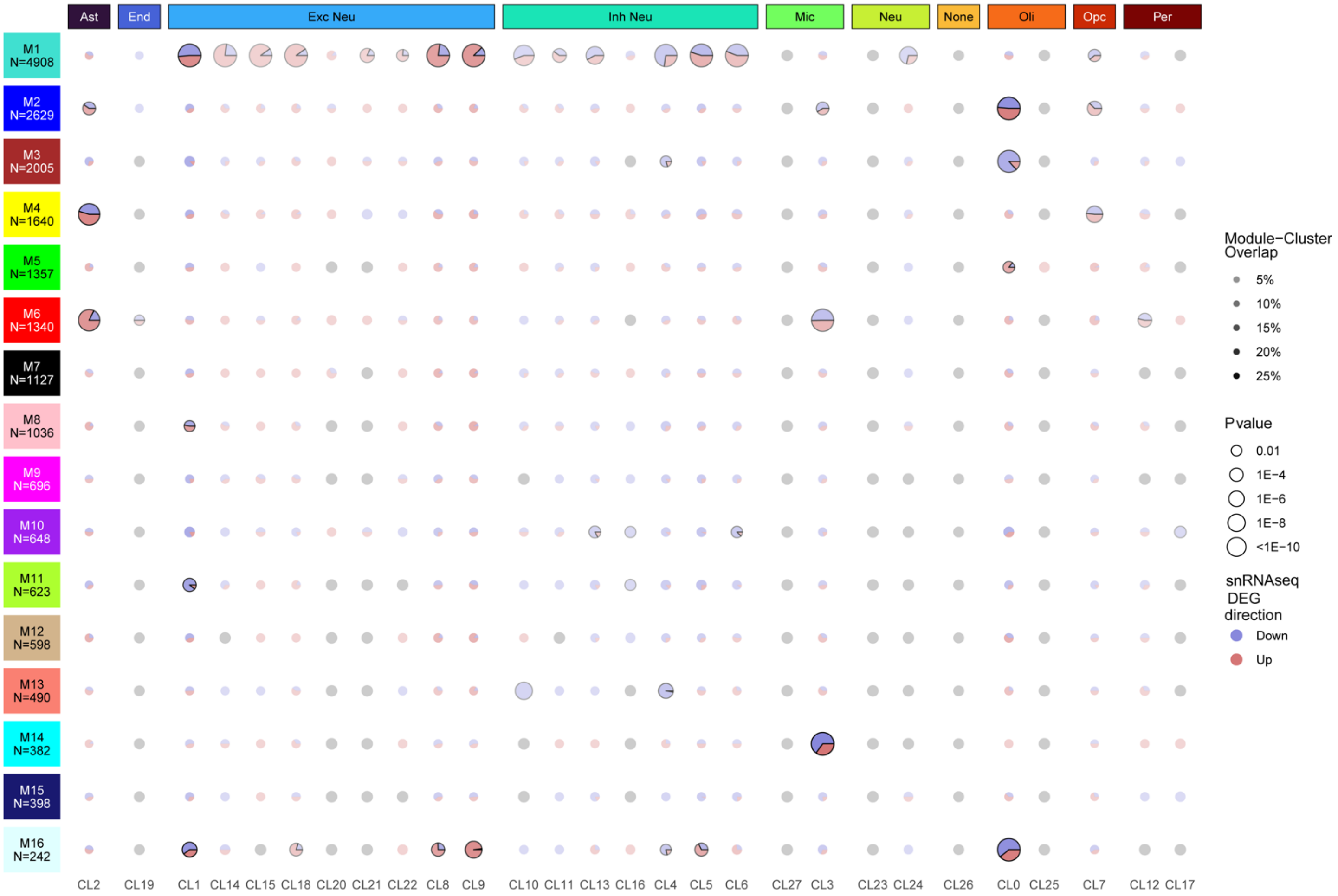

Figure S14. Overlap of WGCNA module genes and snRNASeq DEG genes. The number of overlapping genes between snRNAseq DEGs in each cluster and the 16 WGCNA modules. P value: Fisher's exact test for the enrichment of snRNAseq DEGs in the expression module. Pie chart demonstrates proportion of up- and down-regulated genes. The radii of the pie charts reflect the significance of the overlap, where significant overlaps ($p < 0.05$) have solid outline. Transparency of pie chart reflects the size of the overlap between module genes and cluster DEGs (more opaque=higher number of overlapping genes). Statistics: one-sided Fisher Exact test. Source data are provided as a Source Data file.

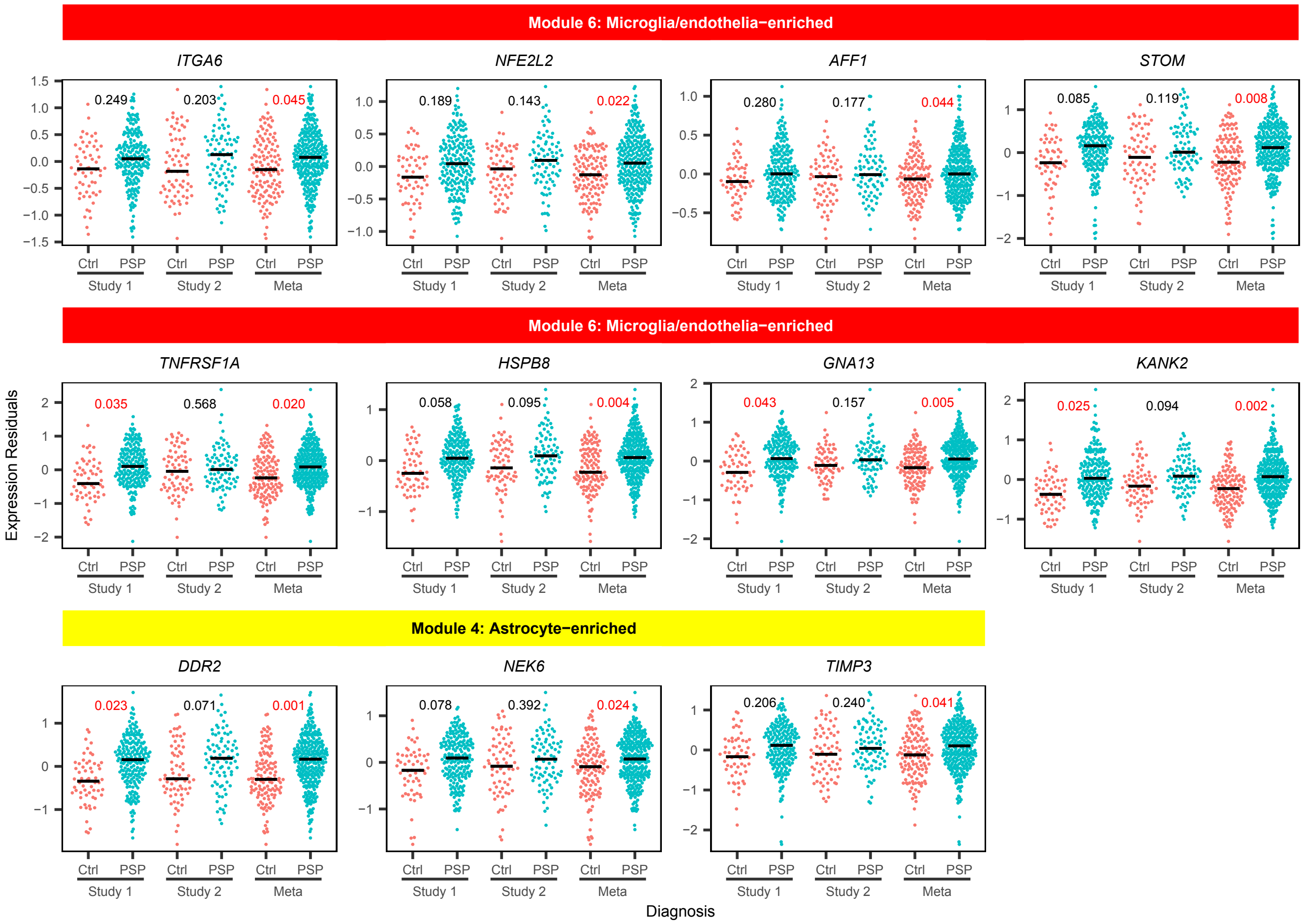

Figure S15. Expression level of the Drosophila tau model screened genes between PSP and control in bulk RNAseq. Statistics: differential expression analysis using linear regression adjusting for covariates. FDR-adjusted p values are presented. Unadjusted p values are provided in Supplementary Data 1 and source data. Study 1: N = 257 PSP and Control individuals; Study 2: N = 151 PSP and Control individuals; Meta: N = 408 PSP and Control individuals. Red text indicates up-regulation in PSP. Blue text indicates down-regulation in PSP.

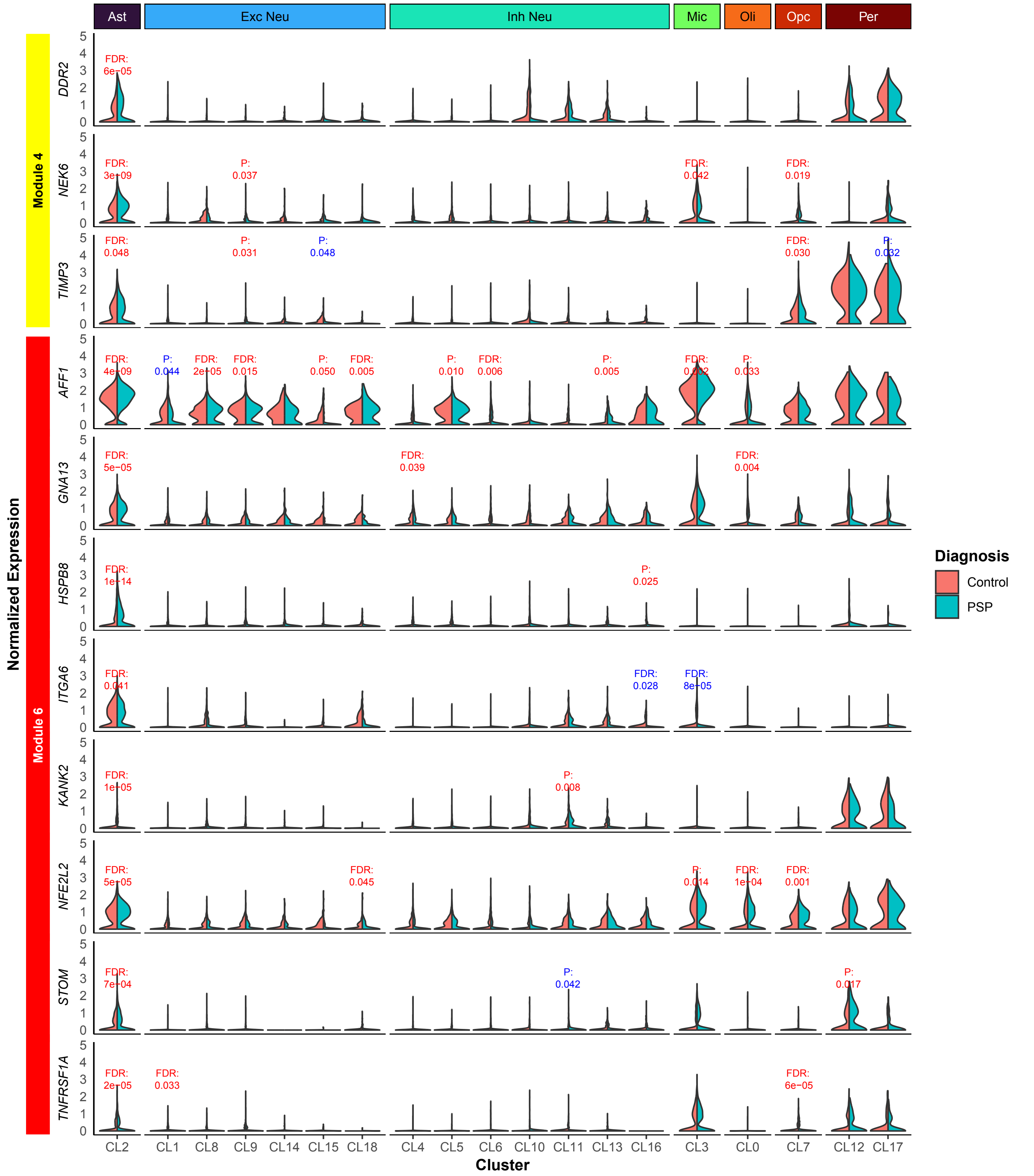

Statistics test: Zero−inflated regression adjusting for sex and age, implemented by the MAST package.
Red indicates up−regulation in PSP. Blue indicates down−regulation in PSP.
Only show clusters with more than 1% of the total nuclei

Figure S16. Expression levels of the Drosophila tau model screened genes between PSP and control in snRNAseq. Statistics: Zero-inflated generalized linear regression comparing PSP vs Control. nuclei adjusting for sex and age, implemented by the MAST package. Significance: FDR-adjusted (FDR) or unadjusted (P) p values as indicated. Red FDR/p indicates up-regulation in PSP. Blue FDR/p indicates

down-regulation in PSP. Expression changes with unadjusted p values greater than 0.05 were not annotated for clarity.

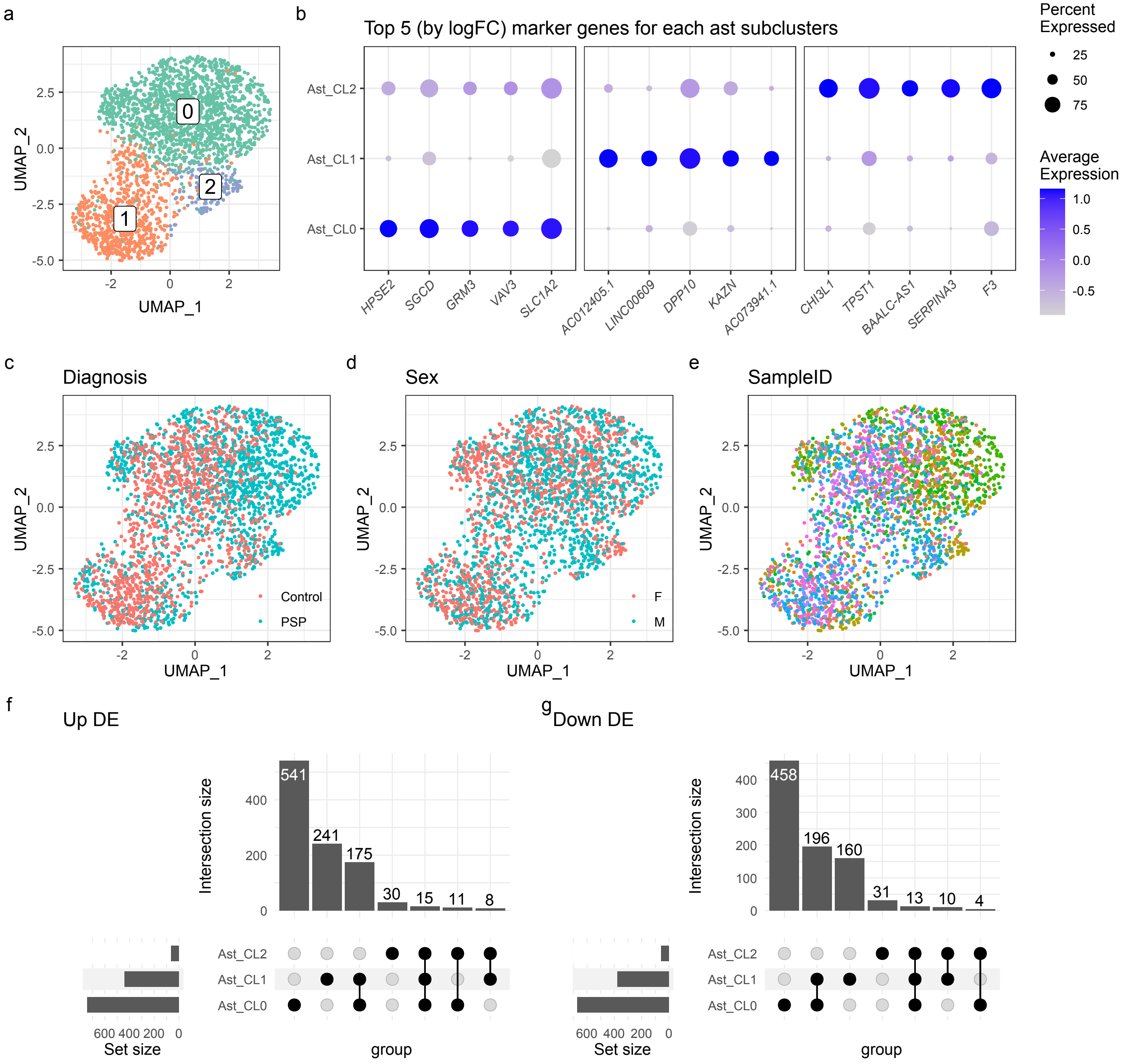

h

GeneName	subcluster	PSPvsCtrl			Control		PSP	
		logFC	pvalue	FDR	avg_exp	pct_exp	avg_exp	pct_exp
DDR2	Ast_CL0	0.0869	0.09324	0.2708	0.6963	58.73%	1.09721	59.12%
	Ast_CL1	0.2124	0.00021	0.0051	0.7960	46.34%	1.09811	60.42%
	Ast_CL2	-0.0262	0.46701	0.8701	0.8575	64.06%	0.84830	70.34%
KANK2	Ast_CL0	0.0343	0.00401	0.0277	0.2504	25.09%	0.32264	26.17%
	Ast_CL1	0.0281	0.00270	0.0330	0.2060	15.30%	0.30969	27.56%
	Ast_CL2	0.0771	0.46194	0.8683	0.2039	26.56%	0.26378	38.98%
STOM	Ast_CL0	0.0614	0.02658	0.1138	0.5195	55.27%	0.57767	48.23%
	Ast_CL1	0.0995	0.01504	0.1064	0.4368	35.34%	0.61523	54.42%
	Ast_CL2	0.2406	0.01463	0.2901	0.4465	53.12%	0.76158	75.42%

Figure S17. Subclustering analysis of the astrocyte nuclei. **a**: UMAP projection of the nuclei and the subcluster assignment. **b**: Top 5 marker genes for each subcluster. **c-e**: UMAP projection of the nuclei colored by key covariates. **f-g**: The number of significant PSP vs Control DEGs in each cluster and their overlaps. Interestingly, there are many overlaps between the DEGs in Ast_CL0 and Ast_CL1 in both directions. **h**: PSP vs Control DEG statistics for *DDR2*, *KANK2*, and *STOM*. Source data are provided as a Source Data file.

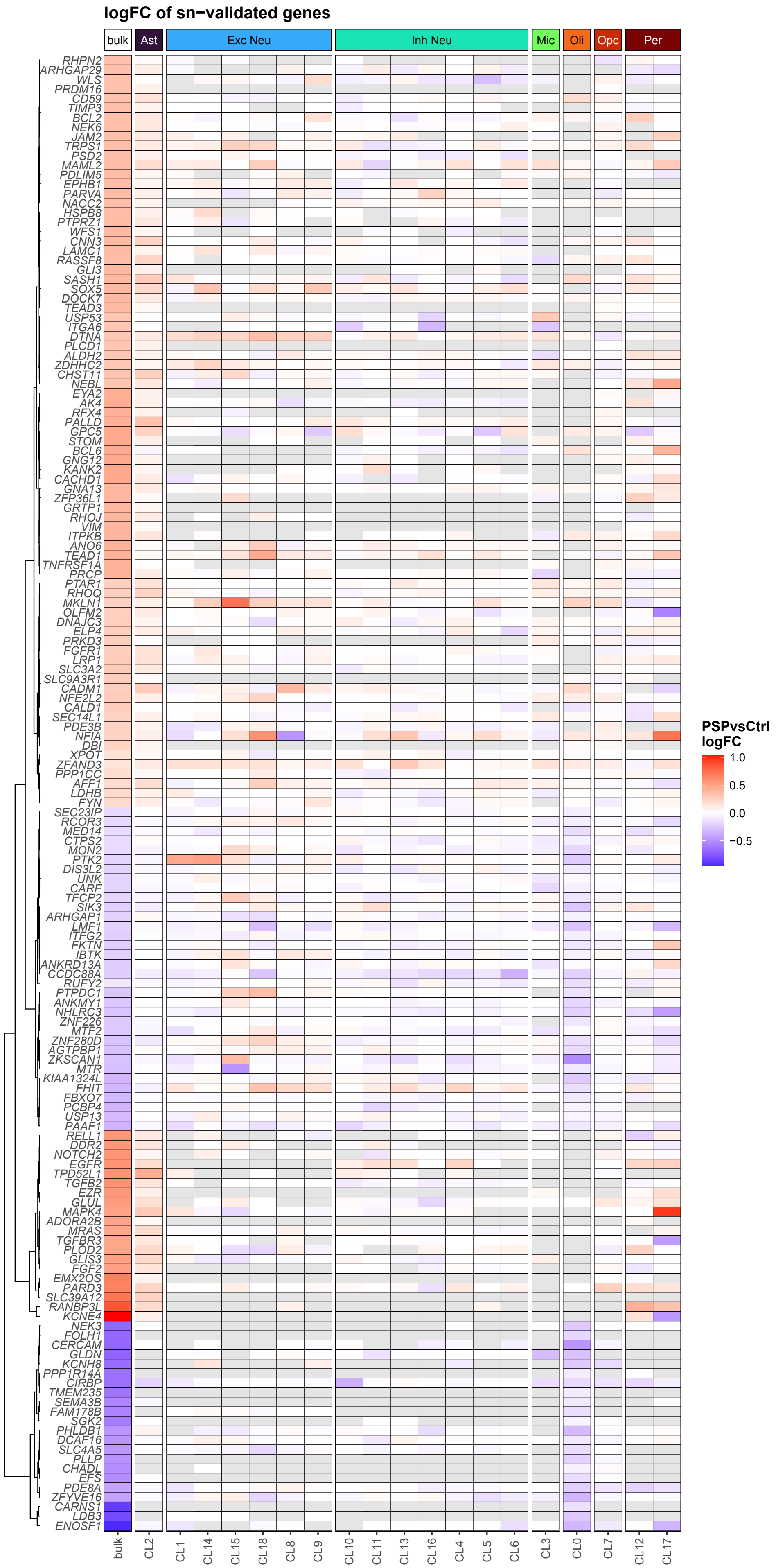
logFC of sn−validated genes
bulk
Ast
Exc Neu
Inh Neu
Mic
Oli
Opc
Per
RHPN2
ARHGAP29
WLS
PRDM16
CD59
TIMP3
BCL2
NEK6
JAM2
TRPS1
PSD2
MAML2
PDLIM5
EPHB1
PARVA
NACC2
HSPB8
PTPRZ1
WFS1
CNN3
LAMC1
RASSF8
GLI3
SASH1
SOX5
DOCK7
TEAD3
USP53
ITGA6
DTNA
PLCD1
ALDH2
ZDHHC2
CHST11
NEBL
EYA2
AK4
RFX4
PALLD
GPC5
STOM
BCL6
GNG12
KANK2
CACHD1
GNA13
ZFP36L1
GRTP1
RHOJ
VIM
ITPKB
ANO6
TEAD1
TNFRSF1A
PRCP
PTAR1
RHOQ
MKLN1
OLFM2
DNAJC3
ELP4
PRKD3
FGFR1
LRP1
SLC3A2
SLC9A3R1
CADM1
NFE2L2
CALD1
SEC14L1
PDE3B
NFIA
DBI
XPOT
ZFAND3
PPP1CC
AFF1
LDHB
FYN
SEC23IP
RCOR3
MED14
CTPS2
MON2
PTK2
DIS3L2
UNK
CARF
TFCP2
SIK3
ARHGAP1
LMF1
ITFG2
FKTN
IBTK
ANKRD13A
CCDC88A
RUFY2
PTPDC1
ANKMY1
NHLRC3
ZNF226
MTF2
ZNF280D
AGTPBP1
ZKSCAN1
MTR
KIAA1324L
FHIT
FBXO7
PCBP4
USP13
PAAF1
RELL1
DDR2
NOTCH2
EGFR
TPD52L1
TGFB2
EZR
GLUL
MAPK4
ADORA2B
MRAS
TGFBR3
PLOD2
GLIS3
FGF2
EMX2OS
PARD3
SLC39A12
RANBP3L
KCNE4
NEK3
FOLH1
CERCAM
GLDN
KCNH8
PPP1R14A
CIRBP
TMEM235
SEMA3B
FAM178B
SGK2
PHLDB1
DCAF16
SLC4A5
PLLP
CHADL
EFS
PDE8A
ZFYVE16
CARNS1
LDB3
ENOSF1
bulk
CL2
CL1
CL14
CL15
CL18
CL8
CL9
CL10
CL11
CL13
CL16
CL4
CL5
CL6
CL3
CL0
CL7
CL12
CL17
PSPvsCtrl
logFC
1.0
0.5
0.0
−0.5

Figure S18. Bulk RNAseq log fold change of the 155 sn-validated genes. Source data are provided as a Source Data file.

Example Gating Strategy for Nuclei Isolation

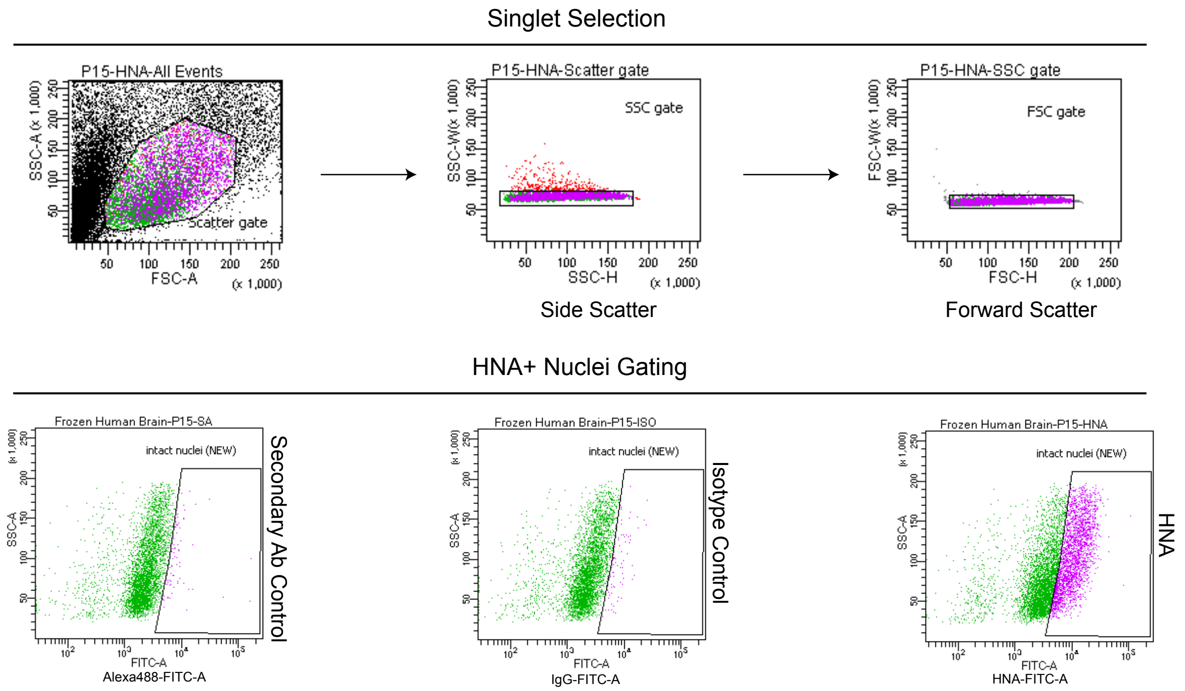

Tube: P15-SA

Population	#Events	%Parent	%Total
All Events	30,000	####	100.0
Scatter gate	4,539	15.1	15.1
SSC gate	4,319	95.2	14.4
FSC gate	4,217	97.6	14.1
intact nuclei (NEW)	90	2.1	0.3

Tube: P15-ISO

Population	#Events	%Parent	%Total
All Events	30,000	####	100.0
Scatter gate	4,213	14.0	14.0
SSC gate	4,005	95.1	13.4
FSC gate	3,896	97.3	13.0
intact nuclei (NEW)	86	2.2	0.3

Tube: P15-HNA

Population	#Events	%Parent	%Total
All Events	30,000	####	100.0
Scatter gate	6,964	23.2	23.2
SSC gate	6,636	95.3	22.1
FSC gate	6,514	98.2	21.7
intact nuclei (NEW)	2,574	39.5	8.6

Figure S19. Gating strategy to obtain intact nuclei from PSP and control brain samples. Example flow plots showing the isolation of nuclei from PSP sample 12031. Statistics shows the number and percent of events within the gate.

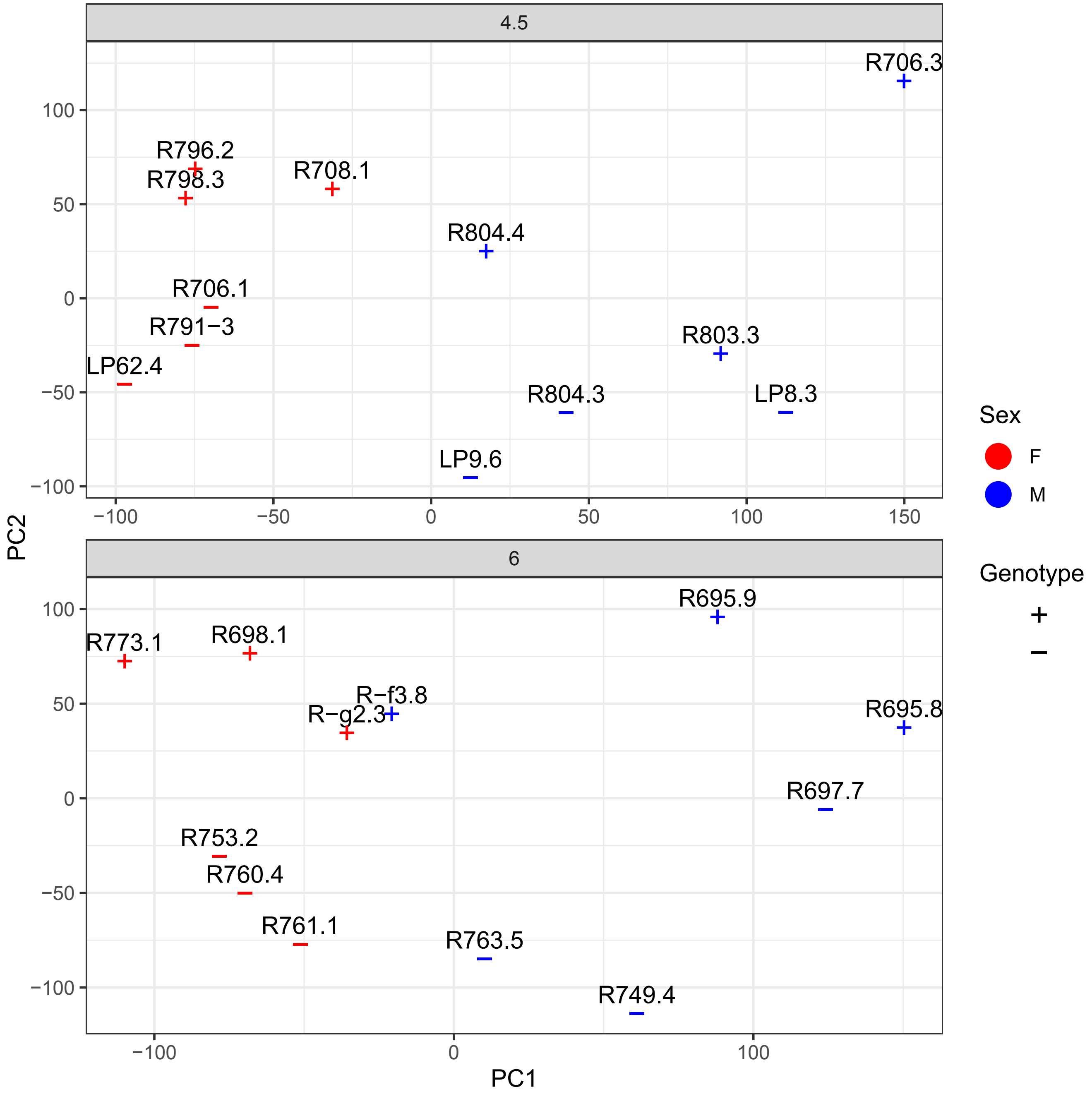

Figure S20: Principal component analysis (PCA) of the mouse expression data. No outlier is detected. Genotype + = rTg4510 mice, - = non-transgenic littermates.

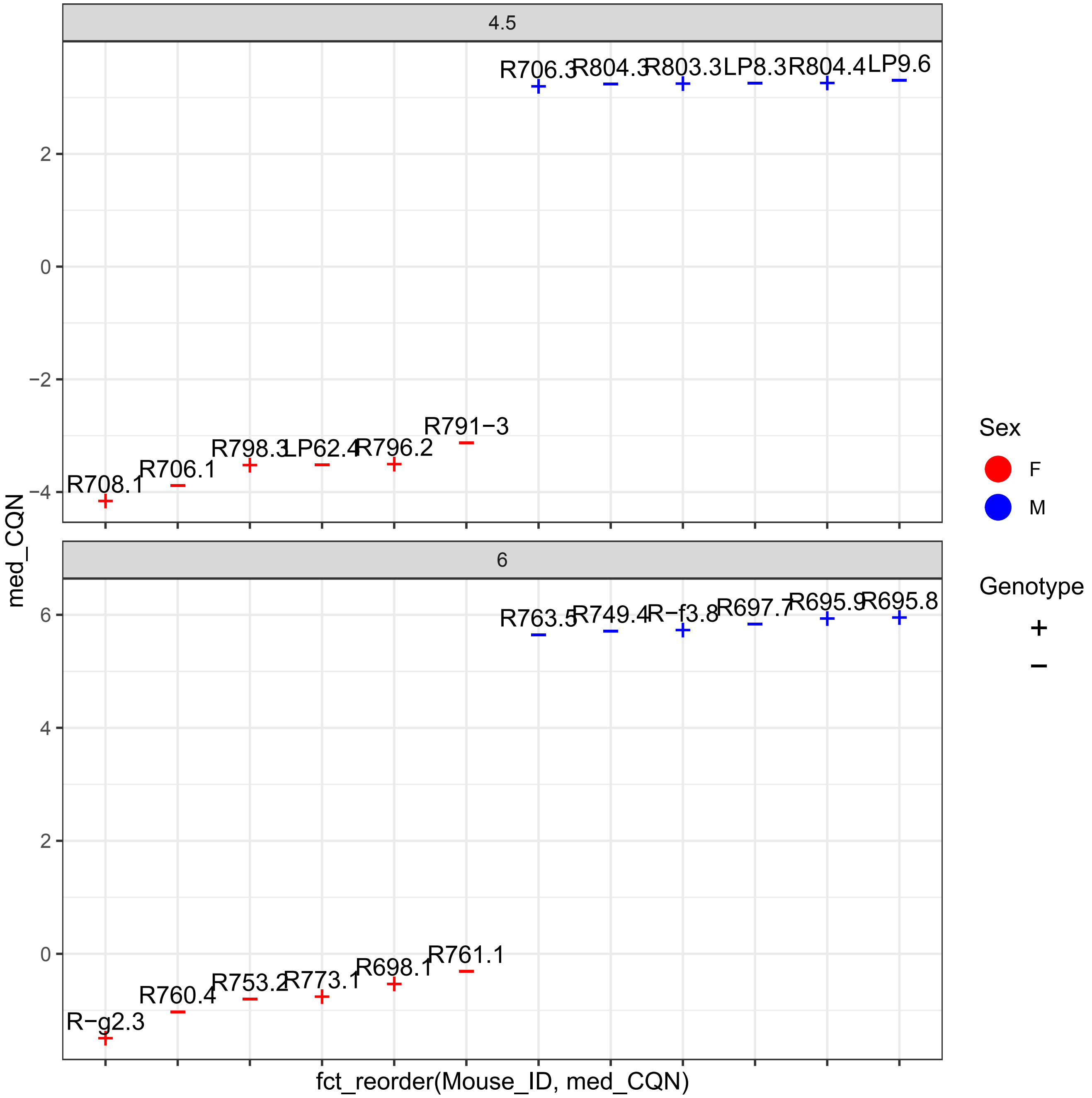

Figure S21: Expression of chromosome Y genes *Ddx3y*, and *Kdm5d* as sex check for the mouse sample. Genotype + = rTg4510 mice, - = non-transgenic littermates.

	Study 1				Study 2			
	**Ctrl**, N = 58[1]	**PSP**, N = 199[1]	**Overall**, N = 257[1]	**p-value**[2]	**Ctrl**, N = 69[1]	**PSP**, N = 82[1]	**Overall**, N = 151[1]	**p-value**[2]
**Sex**				0.856				0.266
Female	34 (59%)	114 (57%)	148 (58%)		34 (49%)	33 (40%)	67 (44%)	
Male	24 (41%)	85 (43%)	109 (42%)		35 (51%)	49 (60%)	84 (56%)	
**Age**	84 (79, 92)	78 (71, 83)	79 (73, 84)	2e-06	86 (78, 89)	74 (69, 79)	78 (72, 85)	3e-10
**RIN**	8.80 (8.00, 9.20)	8.10 (7.30, 8.90)	8.30 (7.50, 9.00)	0.002	7.90 (7.10, 8.50)	8.40 (8.10, 8.80)	8.30 (7.90, 8.65)	2e-06
**Source**				2e-36				2e-20
Banner	0 (0%)	0 (0%)	0 (0%)		44 (64%)	0 (0%)	44 (29%)	
Mayo	15 (26%)	199 (100%)	214 (83%)		25 (36%)	82 (100%)	107 (71%)	
UKY	43 (74%)	0 (0%)	43 (17%)		0 (0%)	0 (0%)	0 (0%)	

[1]n (%); Median (IQR)

[2]Pearson's Chi-squared test; Wilcoxon rank sum test; Fisher's exact test

Table S1. Demographic information for the bulk RNAseq. Statistical tests are two-sided.

	N = 22,560[1]
Gene Type	
protein_coding	15,151 (67%)
antisense	2,717 (12%)
pseudogene	1,889 (8.4%)
lincRNA	1,619 (7.2%)
processed_transcript	327 (1.4%)
less than 1%	857 (3.8%)

[1] n (%)

Table S2. Type of detected genes. Source data are provided as a Source Data file.

DEG Direction	GeneName	GWAS SNP
Down in PSP	*AARSD1*	rs8070723(*MAPT*), rs242557(*MAPT*)
	ASB16	
	ASB16-AS1	
	MOBP	rs10675541(*MOBP*), rs1768208(*MOBP*)
	PTGES3L	rs8070723(*MAPT*), rs242557(*MAPT*)
	PTGES3L-AARSD1	
	RP5-882C2.2	
	TMEM106A	
	ACBD4	rs71920662(*MAPT*)
	ARHGAP27	
	ATP5G2	rs147124286(*SP1*)
	DND1P1	rs71920662(*MAPT*)
	FAM49B	rs2045091(*ASAP1*)
	KANSL1-AS1	rs71920662(*MAPT*)
	LRRC37A	
	MAP3K14-AS1	
	RP11-259G18.3	
	RP11-351D16.3	rs2142991(*BMS1*)
	RP11-707O23.5	rs71920662(*MAPT*)
	TENC1	rs147124286(*SP1*)
	ZNF37BP	rs2142991(*BMS1*)
Up in PSP	*COASY*	rs8070723(*MAPT*), rs242557(*MAPT*)
	CTD-3199J23.4	
	ETV4	
	KCNJ8	rs7966334(*SLCO1A2*), rs11568563(*SLCO1A2*)
	LDHB	
	MYRIP	rs10675541(*MOBP*), rs1768208(*MOBP*)
	PSME3	rs8070723(*MAPT*), rs242557(*MAPT*)
	RND2	
	RP11-284H19.1	rs7966334(*SLCO1A2*), rs11568563(*SLCO1A2*)
	RP11-424C20.2	
	RP11-59N23.1	
	RP11-729I10.2	
	Y_RNA	rs8070723(*MAPT*), rs242557(*MAPT*)
	ADCY8	rs2045091(*ASAP1*)
	CBX5	rs147124286(*SP1*)
	HEXIM1	rs71920662(*MAPT*)
	IARS2	rs6687758(None)
	MORF4L1P1	
	RP11-33M22.2	rs57113693(*STX6*)
	RP11-737F9.1	rs2045091(*ASAP1*)
	RP11-793H13.8	rs147124286(*SP1*)

Table S3. PSP DEGs that are within 1Mbp of PSP GWAS loci.

	**Control**, N = 16[1]	**PSP**, N = 18[1]	**Overall**, N = 34[1]	**p-value**[2]
**RIN**	7.85 (6.47, 8.22)	7.55 (6.90, 7.80)	7.70 (6.73, 8.17)	0.972
**Sex**				0.492
Female	9 (56%)	8 (44%)	17 (50%)	
Male	7 (44%)	10 (56%)	17 (50%)	
**Age**	90 (87, 93)	86 (82, 91)	89 (83, 92)	0.104
**Source**				
Mayo	16 (100%)	18 (100%)	34 (100%)	

[1]Median (IQR); n (%)

[2]Wilcoxon rank sum test; Pearson's Chi-squared test

Table S4. Demographic information for the snRNAseq. Statistical tests are two-sided.

Cell Type	Cluster	Up	Down	N.S.
Astrocyte	CL2	1,477	1,121	28,240
Endothelia	CL19	1	4	30,833
Excitatory neuron	CL22	34	14	30,790
	CL21	23	14	30,801
	CL20	20	5	30,813
	CL18	282	78	30,478
	CL15	99	21	30,718
	CL14	104	71	30,663
	CL9	1,946	534	28,358
	CL8	1,451	725	28,662
	CL1	1,354	2,260	27,224
Inhibitory neuron	CL16	12	19	30,807
	CL13	133	260	30,445
	CL11	47	41	30,750
	CL10	40	60	30,738
	CL6	403	484	29,951
	CL5	658	996	29,184
	CL4	270	638	29,930
Microglia	CL27	0	0	30,838
	CL3	537	659	29,642
Neuron	CL24	22	37	30,779
	CL23	0	0	30,838
None	CL26	0	0	30,838
Oligodendrocyte	CL25	1	0	30,837
	CL0	1,293	1,242	28,303
OPC	CL7	440	412	29,986
Pericyte	CL17	5	4	30,829
	CL12	44	35	30,759

Table S5. Number of DEG (PSP vs Ctrl) in each snRNAseq cluster.

	**Control**, N = 12[1]	**Transgenic**, N = 12[1]	**Overall**, N = 24[1]	**p-value**[2]
**RIN**	7.20 (6.90, 7.50)	7.20 (7.07, 7.62)	7.20 (7.07, 7.53)	0.663
**Sex**				1.000
Female	6 (50%)	6 (50%)	12 (50%)	
Male	6 (50%)	6 (50%)	12 (50%)	
**Age (months)**				1.000
4.5	6 (50%)	6 (50%)	12 (50%)	
6	6 (50%)	6 (50%)	12 (50%)	

[1]Median (IQR); n (%)

[2]Wilcoxon rank sum test; Pearson's Chi-squared test

Table S6. Mouse demographic information. Statistical tests are two-sided.

Gene	Study	Simple			Comprehensive		
		logFC	pvalue	FDR	logFC	pvalue	FDR
DDR2	study 1	0.539	9e-04	0.023	0.208	5e-05	3e-04
	study 2	0.599	0.005	0.071	0.226	0.002	0.010
	meta	0.561	1e-05	0.001	0.213	2e-07	3e-06
STOM	study 1	0.431	0.010	0.085	0.137	0.008	0.022
	study 2	0.507	0.015	0.119	0.124	0.061	0.131
	meta	0.461	4e-04	0.008	0.132	0.001	0.004
KANK2	study 1	0.523	0.001	0.025	0.341	2e-07	3e-06
	study 2	0.416	0.009	0.094	0.304	1e-04	0.001
	meta	0.469	3e-05	0.002	0.326	3e-11	1e-09

Table S7. PSP vs Control DEG statistics for the top 3 candidate genes in the Simple model (not adjusting for cell proportion) and the Comprehensive model (adjusting for cell proportion). Statistics: Linear regression compares PSP and control gene expression adjusting for covariates. Study 1: N = 257 PSP and Control individuals; Study 2: N = 151 PSP and Control individuals; Meta: N = 408 PSP and Control individuals. FDR-corrected p values are also provided.

Gene	Cell	Cluster	MAST			Pseudobulk		
			logFC	pvalue	FDR	logFC	pvalue	FDR
DDR2	oli	CL0	NA	NA	NA	0.111	0.763	0.936
	ast	CL2	0.139	6e-06	6e-05	0.361	0.089	0.498
	mic	CL3	NA	NA	NA	-0.070	0.919	1.000
	opc	CL7	NA	NA	NA	-0.136	0.749	0.970
	per	CL12	0.018	0.774	0.968	-0.077	0.743	0.969
		CL17	-0.077	0.409	1.000			
KANK2	oli	CL0	NA	NA	NA	1.159	0.007	0.160
	ast	CL2	0.047	1e-06	1e-05	0.726	0.013	0.236
	mic	CL3	NA	NA	NA	0.023	0.962	1.000
	opc	CL7	NA	NA	NA	0.371	0.497	0.912
	per	CL12	0.033	0.855	0.984	-0.241	0.333	0.864
		CL17	0.036	0.090	0.886			
STOM	oli	CL0	NA	NA	NA	1.221	0.003	0.107
	ast	CL2	0.087	1e-04	7e-04	0.435	0.010	0.208
	mic	CL3	0.084	0.081	0.201	0.509	0.018	0.497
	opc	CL7	NA	NA	NA	0.194	0.691	0.963
	per	CL12	0.081	0.017	0.302	0.538	0.033	0.634
		CL17	0.028	0.757	1.000			

Table S8. PSP vs Control DE results using MAST in each cluster and pseudobulk in each cell type for the top nominated genes. Statistics: MAST: Zero-inflated regression comparing PSP vs Control nuclei adjusting for sex and age, implemented by the MAST package. Pseudobulk: Negative binomial generalized linear model comparing aggregated gene count between PSP vs control at individual-level. FDR-corrected p values are also provided.

Human Symbol	Fly GeneID	FlyBaseID	Fly Symbol
AFF1	33496	FBgn0041111	lilli
DDR2	3346209	FBgn0053531	Ddr
GNA13	3355131	FBgn0000384	cta
HSPB8	32955	FBgn0031037	CG14207
ITGA6	32275	FBgn0004456	mew
KANK2	36668	FBgn0027596	Kank
NEK6	31696	FBgn0029970	Nek2
NFE2L2	42743	FBgn0262975	cnc
STOM	38562	FBgn0260657	CG42540
TIMP3	41248	FBgn0025879	Timp
TNFRSF1A	37216	FBgn0086358	Tab2

Table S9: *Drosophila* ortholog genes.

BDSC ID	Note
26314	lilli RNAi
55906	Ddr RNAi
31132	cta RNAi
64571	CG14207 RNAi
27543	mew RNAi
33432	Kank RNAi
28600	Nek2 RNAi
25984	cnc RNAi
65975	CG42540 RNAi
61294	Timp RNAi
29417	Tab2 RNAi
51363	UAS-Tau
9146	GMR-Gal4
3703	Balancer
3605	w[1118] wildtype
64349	Canton-S wildtype

Table S10: *Drosophila* lines used and their ID from the Bloomington Drosophila Stock Center.

Supplementary References

1 Jiang, J., Wang, C., Qi, R., Fu, H. & Ma, Q. scREAD: A Single-Cell RNA-Seq Database for Alzheimer's Disease. *iScience* **23**, 101769 (2020). https://doi.org:10.1016/j.isci.2020.101769

2 McKenzie, A. T. *et al.* Brain Cell Type Specific Gene Expression and Co-expression Network Architectures. *Sci Rep-Uk* **8** (2018). https://doi.org:ARTN 8868
10.1038/s41598-018-27293-5
