# Supplementary material for: Cross species systems biology discovers glial DDR2, STOM, and KANK2 as therapeutic targets in progressive supranuclear palsy
Source: Nat Commun. 2023 Nov 2;14:6801. doi: 10.1038/s41467-023-42626-3 (PMC10622416; doi:10.1038/s41467-023-42626-3)
Supplement: Supplementary file 3 — Description of Additional Supplementary Files [file 41467_2023_42626_MOESM3_ESM.pdf]

Description of Additional Supplementary Files

File Name: Supplementary Data 1
Description: Differential gene expression analysis results using the bulk RNAseq data

File Name: Supplementary Data 2
Description: WGCNA module eigengenes, module membership, enrichment of cell type marker genes, and correlation with different phenotypes

File Name: Supplementary Data 3
Description: Enriched gene ontology terms in each WGCNA module

File Name: Supplementary Data 4
Description: : Significant differentially expressed genes between PSP and control either at the cluster level using MAST or at a cell type level using a pseudobulk approach based on the snRNAseq dataset

File Name: Supplementary Data 5
Description: Small molecules and gene interaction status, retrieved from DGIdb
