# Supplementary material for: Cross species systems biology discovers glial DDR2, STOM, and KANK2 as therapeutic targets in progressive supranuclear palsy
Source: Nat Commun. 2023 Nov 2;14:6801. doi: 10.1038/s41467-023-42626-3 (PMC10622416; doi:10.1038/s41467-023-42626-3)
Supplement: Supplementary file 9 — Reporting Summary [file 41467_2023_42626_MOESM9_ESM.pdf]

nature portfolio

Corresponding author(s): Nilufer Ertekin-Taner

Last updated by author(s): Sep 18, 2023

Reporting Summary

Nature Portfolio wishes to improve the reproducibility of the work that we publish. This form provides structure for consistency and transparency in reporting. For further information on Nature Portfolio policies, see our Editorial Policies and the Editorial Policy Checklist.

Statistics

For all statistical analyses, confirm that the following items are present in the figure legend, table legend, main text, or Methods section.

n/a	Confirmed	
☐	☒	The exact sample size (*n*) for each experimental group/condition, given as a discrete number and unit of measurement
☐	☒	A statement on whether measurements were taken from distinct samples or whether the same sample was measured repeatedly
☐	☒	The statistical test(s) used AND whether they are one- or two-sided<br>*Only common tests should be described solely by name; describe more complex techniques in the Methods section.*
☐	☒	A description of all covariates tested
☐	☒	A description of any assumptions or corrections, such as tests of normality and adjustment for multiple comparisons
☐	☒	A full description of the statistical parameters including central tendency (e.g. means) or other basic estimates (e.g. regression coefficient) AND variation (e.g. standard deviation) or associated estimates of uncertainty (e.g. confidence intervals)
☐	☒	For null hypothesis testing, the test statistic (e.g. *F*, *t*, *r*) with confidence intervals, effect sizes, degrees of freedom and *P* value noted<br>*Give P values as exact values whenever suitable.*
☒	☐	For Bayesian analysis, information on the choice of priors and Markov chain Monte Carlo settings
☒	☐	For hierarchical and complex designs, identification of the appropriate level for tests and full reporting of outcomes
☐	☒	Estimates of effect sizes (e.g. Cohen's *d*, Pearson's *r*), indicating how they were calculated

Our web collection on statistics for biologists contains articles on many of the points above.

Software and code

Policy information about availability of computer code

Data collection

The following softawres were used for bulk RNA sequencing:
- HCS v3.3.20 collection software
- Illumina's RTA v 2.5.2

The fly eye pictures were taken and processed with Zeiss ZEN Software version 3.0

Data analysis

All analysis was performed using R programming language version 4.0.3 and 4.1.2. Analysis code will be made public after publication.

The following R package/pipelines were used:
- MAP-Rseq pipeline v2.0
- cqn v1.36.0
- sva 3.38.0
- meta 6.0-0
- WGCNA v1.70-3
- anRichment v1.19-3
- Rtsne v 0.16
- Cell Ranger v4.0
- Seurat v4.0.5
- MAST 1.16.0
- edgeR 3.32.1
- biomaRt v2.46.3 with Ensembl version 105

- FUMA GWAS v1.4.0

For visualization, the following R pacakges (versions) were used:
- gtsummary v1.4.2
- ggplot2 v3.4.0
- ggrepel v0.9.1
- ggtext v0.1.2
- ggh4x v0.2.0
- patchwork v1.1.2
- ggpointpie v0.0.2
- ComplexUpset v1.3.0
- rstatix v0.7.0
- circlize v0.4.15
- ComplexHeatmap v2.6.2

For manuscripts utilizing custom algorithms or software that are central to the research but not yet described in published literature, software must be made available to editors and reviewers. We strongly encourage code deposition in a community repository (e.g. GitHub). See the Nature Portfolio guidelines for submitting code & software for further information.

Data

Policy information about availability of data

All manuscripts must include a data availability statement. This statement should provide the following information, where applicable:

- Accession codes, unique identifiers, or web links for publicly available datasets
- A description of any restrictions on data availability
- For clinical datasets or third party data, please ensure that the statement adheres to our policy

All human and mouse RNAseq data in this manuscript is available via the AD Knowledge Portal (https://adknowledgeportal.synapse.org). The AD Knowledge Portal is a platform for accessing data, analyses and tools generated by the Accelerating Medicines Partnership (AMP AD) Target Discovery Program and other National Institute on Aging (NIA)-supported programs to enable open-science practices and accelerate translational learning. Data is available for general research use according to the following requirements for data access and data attribution (https://adknowledgeportal.synapse.org/DataAccess/Instructions). An overview of all the data generated and used in this study can be found on the manuscript landing page (https://doi.org/10.7303/syn51361408). The bulk brain and single-nucleus RNAseq data generated in this study have been deposited in the AD Knowledge Portal under the Mayo RNAseq study (accession ID: syn5550404) [https://doi.org/10.7303/syn5550404] and the Mayo Clinic Brain Molecular Signatures of Alzheimer's Disease (MC-BrAD) study (accession ID: syn51298412) [https://doi.org/10.7303/syn2580853]. The rTG4510 mouse RNAseq data used in this study are available in the AD Knowledge Portal under the Tau and APP mouse model (TAUAPPms) study (accession ID: syn3157182) [https://doi.org/10.7303/syn3157182]. All summary results for human bulk RNAseq PSP DEG, neuropathology associations, WGCNA analysis, snRNAseq, rTG4510 brain associations, Drosophila tau model results, and any available drugs against the prioritized genes based on the Drug Gene Interaction Database (DGIdb) v4.063 is available through our web application (https://rtools.mayo.edu/PSP_RNAseq_Atlas/) and the Supplementary Information. Source data are provided with this paper.

Research involving human participants, their data, or biological material

Policy information about studies with human participants or human data. See also policy information about sex, gender (identity/presentation), and sexual orientation and race, ethnicity and racism.

Reporting on sex and gender	The study cohorts contain balanced proportions of males and females (193, or 47% males in the bulk RNAseq dataset, 17, or 50% males in the single nucleus RNAseq dataset). The study design does not favor one sex over another. The sex information of passed-QC samples is based on both self-reporting and estimation from RNAseq data. De-identified, individual-level metadata is available for general research use according to the following requirements for data access and data attribution (https://adknowledgeportal.synapse.org/DataAccess/Instructions). Samples of both sexes were analyzed jointly with sex as a covariate, and the results from this study are applicable to both sexes.
Reporting on race, ethnicity, or other socially relevant groupings	All participants are Non-Hispanic White (NHW). Socially relevant groupings were not included in the analysis.
Population characteristics	Samples from 419 NHW participants were included in this project. Among the 419 participants, 283 (68%) are PSP cases, 198 (47%) are male. The median age (IQR) for the population is 79 (73, 85). The 408 participants whose samples underwent bulk RNAseq analysis were generated as two independent studies. Study 1 has 257 participants, 199 (77%) are PSP cases, 109 (42%) are male. The median age (IQR) is 79 (73, 84). Study 2 has 151 participants, 82 (54%) are PSP cases, 84 (56%) are male. The median age (IQR) is 78 (72, 85). The single-nucleus RNAseq has 34 participants, 18 (53%) are PSP cases, 17 (50%) are male. The median age (IQR) is 89 (83, 92).
Recruitment	Written informed consent was obtained from all participants or their qualified caregivers. All PSP participants were recruited from Mayo Clinic Brain Bank, while control participants were from Mayo Clinic Brain Bank, the University of Kentucky, or the Banner Sun Health Research Institute.
Ethics oversight	This study was approved by the Mayo Clinic Institutional Review Board (IRB). The use of samples from the University of Kentucky and Banner Sun Health Research Institute has been approved by the appropriate review boards. Additional data used in this study from the AD Knowledge Portal (https://adknowledgeportal.synapse.org) were accessed under the data usage agreement (https://adknowledgeportal.synapse.org/DataAccess/DataUseCertificates). All personally identifiable

information has been removed. Written informed consent was obtained from all participants, their qualified caregivers or next of kin.

Note that full information on the approval of the study protocol must also be provided in the manuscript.

Field-specific reporting

Please select the one below that is the best fit for your research. If you are not sure, read the appropriate sections before making your selection.

☒ Life sciences ☐ Behavioural & social sciences ☐ Ecological, evolutionary & environmental sciences

For a reference copy of the document with all sections, see nature.com/documents/nr-reporting-summary-flat.pdf

Life sciences study design

All studies must disclose on these points even when the disclosure is negative.

Sample size	The human participant sample size was determined by the number of available data. The Mouse RNAseq study sample size was determined by the number of available data. The number of fly used to generate the semiquantitative score is determined based on prior publications (Zhang et al, 2015, doi:10.1038/nature14973)
Data exclusions	For bulk RNAseq study 1, 306 samples were sequenced and six samples failed QC (gene body coverage or sex-mismatch). These six failed samples were not included in the analysis. Additionally, 41 samples with a diagnosis other than PSP or control were excluded. Two samples were obtained from a different brain bank from the other samples and were also excluded from analysis.<br>For bulk RNAseq study 2, 11 samples were excluded, as reported by Allen et al, 2016, https://doi.org/10.1038/sdata.2016.89<br>For snRNAseq, 2 samples failed QC. One sample had a low number of nuclei; the other sample appeared to be an outlier.
Replication	We performed analyses in cohorts from different species and technologies, including two independent human bulk RNAseq cohorts, one human single-nucleus RNAseq cohort, one tau mouse bulk RNAseq cohort, and Drosophila validation. Our top genes showed a consistent effect in the above data sources. All experiments were performed with biologically independent samples except for a small overlap of bulk RNAseq and snRNAseq samples. Specifically, 16 PSP and 7 Control samples in the snRNAseq dataset were also sequenced as part of the bulk RNAseq datasets. For the evaluation of the Drosophila eye phenotype, three independent crosses were set up, each time with qualitative evaluations of the eye phenotype. Complete and blinded quantification of the eye phenotype was performed once.
Randomization	For human patients and controls (human bulk RNAseq and single nucleus RNAseq), randomization was performed such that each batch or flowcell contained a balanced proportion of males/females and diagnosis groups. For the mouse RNAseq data, randomization was performed such that there is an equal number of transgenic and non-transgenic mice of each sex and age. For all analyses, we included relevant covariates such as sex and batch in the model where appropriate. For experiments with drosophila, progenies with the correct genotype, as indicated by the presence/absence of visible mutations (CyO and TM3, Sb1), are selected without any other requirement. In this case, randomization is not relevant.
Blinding	The investigators were blinded when assessing the drosophila eye phenotype by using a unique ID for each fly genotype. For the RNA sequencing experiments, samples were randomized and were assigned a unique identifier. The investigators were blinded in the workflow except for nuclei sorting in snRNAseq data generation, where the diagnosis of the specimen was known. The investigator was not blinded for the analysis of the data.

Reporting for specific materials, systems and methods

We require information from authors about some types of materials, experimental systems and methods used in many studies. Here, indicate whether each material, system or method listed is relevant to your study. If you are not sure if a list item applies to your research, read the appropriate section before selecting a response.

Materials & experimental systems

n/a	Involved in the study	
☐	☒	Antibodies
☒	☐	Eukaryotic cell lines
☒	☐	Palaeontology and archaeology
☐	☒	Animals and other organisms
☒	☐	Clinical data
☒	☐	Dual use research of concern
☒	☐	Plants

Methods

n/a	Involved in the study	
☒	☐	ChIP-seq
☐	☒	Flow cytometry
☒	☐	MRI-based neuroimaging

Antibodies

Antibodies used	We utilized mouse anti-Human Nuclear Antigen [235-1] (ab191181, Abcam, 1:50), Mouse IgG1, kappa monoclonal [15-6E10A7] isotype control (ab170190, Abcam, 1:50), and goat anti-mouse Alexa488 secondary antibody (ab150113, Abcam, 1:200).

Validation	Anti-Human Nuclear Antigen was used to track transplanted human cells in other species. We hypothesized that nuclei with compromised membrane may have leakage of histone proteins that causes nuclei to become sticky. For this reason, we gated for HNA+ nuclei and sorted only this population to obtain high quality intact nuclei. Examples of the flow gating are provided in Supplementary Figure 19.

Animals and other research organisms

Policy information about studies involving animals; ARRIVE guidelines recommended for reporting animal research, and Sex and Gender in Research

Laboratory animals	Drosophila melanogaster (genotype information outlined in supplementary data). Quantification of the eye phenotype was performed 5 days after progenies eclose (please also see Methods section).
Wild animals	This study did not involve wild animals.
Reporting on sex	Both sexes were included in the analysis.
Field-collected samples	The study did not involve samples collected from the field.
Ethics oversight	The study was approved by the appropriate institutional review boards.

Note that full information on the approval of the study protocol must also be provided in the manuscript.

Flow Cytometry

Plots

Confirm that:

☒ The axis labels state the marker and fluorochrome used (e.g. CD4-FITC).

☒ The axis scales are clearly visible. Include numbers along axes only for bottom left plot of group (a 'group' is an analysis of identical markers).

☒ All plots are contour plots with outliers or pseudocolor plots.

☒ A numerical value for number of cells or percentage (with statistics) is provided.

Methodology

Sample preparation	Frozen temporal cortex tissue samples were obtained from the Mayo Clinic Brain Bank. Total RNA from ~20 mg collected tissue was isolated to evaluate the quality of tissue. RNA integrity number (RIN) was determined via Agilent 2100 Bioanalyzer using RNA Pico Chip assay, and tissues that have RIN > 6.0 were utilized in nuclei isolation and single nucleus RNA sequencing (snRNAseq).<br>For each participant, 100 mg tissue sample was used for nuclei isolation using a modified protocol47. Samples were homogenized with 25 strokes of loose and tight pestle sequentially using dounce homogenizer in homogenization buffer (0.25 M sucrose, 25 mM KCl, 5 mM MgCl2, 20 mM tricine-KOH, pH 7.8, 1 mM DTT, 0.15 mM spermine, 0.5 mM spermidine, protease inhibitors, 5 µ g/mL actinomycin, 5 u/ µL recombinant RNAase inhibitor, and 0.04% BSA). IGEPAL (5%, Sigma, I8896) solution was added following stroke with the tight pestle to a final concentration of 0.32%. After 10 additional strokes, the tissue homogenate was filtered using a 30 µm cell strainer. Debris was pelleted by centrifugation at 500g for 5 minutes and washed with Wash and Storage Buffer (WSB, 1XPBS with 2%BSA and 5 u/ µL recombinant RNAase inhibitor (Takara Bio, 2313A)). The nuclei-containing supernatant was filtered again with a 30 µm cell strainer, followed by centrifugation at 500g for 10 minutes. After re-suspending the pallet in 700µl cold PBS with 5 U/µl RNAse inhibitors, 300µl debris removal solution (Miltenyi Biotech) was added, and the solution was gently mixed. Another 1 mL WSB was carefully overlaid on top of the nuclei solution. The supernatant was removed after centrifugation at 3000g for 10 minutes. The nuclei were washed once with WSB and pelleted after centrifugation for 10 minutes at 1000g.<br>Isolated nuclei were sorted using fluorescence-activated nuclei sorting (FANS). Human Nuclear Antigen [235-1] (ab191181, Abcam) antibody was applied to the nuclei at 1:50 and incubated for 1 hour on ice. Concurrently, mouse IgG1, kappa monoclonal [15-6E10A7] isotype was included as controls (ab170190, Abcam, 1:50). Goat anti-mouse Alexa488 secondary antibodies (ab150113, Abcam, 1:200) were incubated with the nuclei for 30 minutes on ice. The stained nuclei were reconstituted in WSB and sorted using BD FACSAria II sorter using the 70-micron nozzle with 70 psi sheath pressure and 1.5 ND filter.
Instrument	BD FACS Aria II (BD Biosciences)
Software	FCS files were exported and data was analyzed in FlowJo v10.7
Cell population abundance	The average total number of sorted nuclei per sample is 175,982. The sorted nuclei were run through the flow cytometer again using the established gating criteria to determine the purity and quality of the nuclei samples. The purity of the sorted nuclear samples was determined by comparing post vs pre-sorted HNA+ fractions in the total events. In pre-sorted samples, among all events, an average of 9.6% are HNA+, while in post-sorted samples, among all events, an average of 42.63% are HNA+. Representing an average of 6.48-fold enrichment in HNA+ fraction after sorting, confirming the enrichment of pure nuclei.

Gating strategy

FSC-A/SSC-A gates were set to select nuclear fraction. SSC-H/SSC-W and FSC-H/FSC-W gates were applied to nuclear fraction to discriminate doublets. Secondary antibody stained nuclei control sample were gated for HNA+ in FITC-H/SSC-H channel. This gate was applied to anti-HNA stained sample and sorting was performed. Sorted nuclear sample is run again FACS Aria II to validated the sorting and determine the efficiency of the sorting.

☒ Tick this box to confirm that a figure exemplifying the gating strategy is provided in the Supplementary Information.
